# Phosphorylation Modulates Catalytic Activity of Mycobacterial Sirtuins

**DOI:** 10.3389/fmicb.2016.00677

**Published:** 2016-05-09

**Authors:** Ghanshyam S. Yadav, Sandeep K. Ravala, Neha Malhotra, Pradip K. Chakraborti

**Affiliations:** CSIR-Institute of Microbial TechnologyChandigarh, India

**Keywords:** eukaryotic-type serine/threonine kinases, sirtuin, NAD^+^-dependent deacetylase, protein phosphorylation, enzyme kinetics, site-directed mutagenesis

## Abstract

Sirtuins are NAD^+^-dependent deacetylases involved in the regulation of diverse cellular processes and are conserved throughout phylogeny. Here we report about *in vitro* transphosphorylation of the only NAD^+^-dependent deacetylase (mDAC) present in the genome of *Mycobacterium tuberculosis* by eukaryotic-type Ser/Thr kinases, particularly PknA. The phosphorylated mDAC displayed decreased deacetylase activity compared to its unphosphorylated counterpart. Mass-spectrometric study identified seven phosphosites in mDAC; however, mutational analysis highlighted major contribution of Thr-214 for phosphorylation of the protein. In concordance to this observation, variants of mDAC substituting Thr-214 with either Ala (phospho-ablated) or Glu (phosphomimic) exhibited significantly reduced deacetylase activity suggesting phosphorylation mediated control of enzymatic activity. To assess the role of phosphorylation towards functionality of mDAC, we opted for a sirtuin knock-out strain of *Escherichia coli* (*Δdac*), where interference of endogenous mycobacterial kinases could be excluded. The *Δdac* strain in nutrient deprived acetate medium exhibited compromised growth and complementation with mDAC reversed this phenotype. The phospho-ablated or phosphomimic variant, on the other hand, was unable to restore the functionality of mDAC indicating the role of phosphorylation *per se* in the process. We further over-expressed mDAC or mDAC-T214A as His-tagged protein in *M. smegmatis*, where endogenous eukaryotic-type Ser/Thr kinases are present. Anti-phosphothreonine antibody recognized both mDAC and mDAC-T214A proteins in western blotting. However, the extent of phosphorylation as adjudged by scanning the band intensity, was significantly low in the mutant protein (mDAC-T214A) compared to that of the wild-type (mDAC). Furthermore, expression of PknA in the mDAC complemented *Δdac* strain was able to phosphorylate *M. tuberculosis* sirtuin. The growth profile of this culture in acetate medium was slow compared to that transformed with only vector. On the other hand, use of a kinase dead variant, PknA-K42N instead of PknA, did not display such behavior, which again supported phosphorylation mediated control of mDAC protein. Thus, our results ostensibly render evidence for cross-talk between two distinct post-translational modifications, phosphorylation and deacetylation, in any bacteria. Bioinformatic analysis further indicated conservation of Thr-214 among different mDAC orthologs, thereby arguing the event as mycobacteria specific.

## Introduction

Sirtuins, the NAD^+^-dependent deacetylases are implicated in regulating multitude of cellular processes in eukaryotes that includes energy homeostasis, metabolism, aging, and regulation of transcription factors (Yeung et al., 2004; Guarente, 2007; Ahn et al., 2008; Zhao et al., 2010). Similarly, in prokaryotes there are accumulating evidences that sirtuins play a crucial role in the regulation of short chain fatty acid metabolism, catabolism of aromatic and alicyclic amino acids, etc. (Starai et al., 2003; Crosby et al., 2010). In fact, cellular metabolic status, which depends on NAD^+^/NADH ratio, is also influenced by deacetylase activity (Hipkiss, 2008). Recent studies in eukaryotes indicated that apart from the energy status of the cell, post-translational modification of proteins, such as cAMP/PKA mediated phosphorylation of SIRT1, maintains its tight regulation (Gerhart-Hines et al., 2011). Strikingly, a large number of metabolic enzymes are acetylated in *Escherichia coli* or *Salmonella enterica* (Wang et al., 2010; Zhang et al., 2013) and their deacetylation by CobB, a homolog of eukaryotic sirtuin, helps in maintaining their cellular metabolic status (Starai et al., 2002; Wang et al., 2010). Recently, proteome-wide lysine acetylation in an intracellular pathogen, *Mycobacterium tuberculosis* was mapped (Liu et al., 2014; Xie et al., 2015). However, till date there is no report indicating phosphorylation mediated regulation of any bacterial sirtuin.

Accordingly, we emphasized here on the NAD^+^-dependent deacetylase, Rv1151c (Gu et al., 2009) of *M. tuberculosis* (hereafter referred as mDAC). In fact, *M. tuberculosis* shows remarkable adaptability to the changing environment within the host to become a successful pathogen. Furthermore, within macrophage this bacterium survives on the fatty acids/cholesterol mainly derived from the host (Pandey and Sassetti, 2008) and uses different nutrients depending on the availability. Recently, it was reported that several enzymes involved in fatty acid metabolism are regulated by reversible acetylation (Nambi et al., 2013). Similarly, eukaryotic-type Ser/Thr kinases in *M. tuberculosis* regulate a number of metabolic processes through reversible phosphorylation (Nguyen et al., 2005). Taken together, this study draws its logical inspiration to have an insight on the possibility of recruiting the PTMs like phosphorylation in controlling mDAC activity for versatile metabolic adaptability of *M. tuberculosis.*

Among 11 mycobacterial eukaryotic-type Ser/Thr kinases, PknA and PknB are associated with cell division, growth, and regulation of metabolic processes (Av-Gay and Everett, 2000; Kang et al., 2005; Fernandez et al., 2006; Thakur and Chakraborti, 2006; Pereira et al., 2011). They are essential (Sassetti et al., 2003; Fernandez et al., 2006) in *M. tuberculosis* and present in the same operon near the origin of replication along with the only Ser/Thr phosphatase, PPP. The importance of both these kinases is further highlighted with presence of their homologs in the minimal genome of *M. leprae* (Cole et al., 2001). In fact, regulation of several proteins (Wag31, PbpA, InhA, etc.) by these kinases has already been reported (Kang et al., 2005; Dasgupta et al., 2006; Khan et al., 2010). Besides this, phosphorylation of GarA, a regulator of central carbon metabolism in mycobacteria, and elongation factor Tu by PknB was documented (Villarino et al., 2005; Sajid et al., 2011). PknB also has several other substrates (Pereira et al., 2011), including many mycobacterial eukaryotic-type Ser/Thr kinases and therefore is believed as a master regulator (Baer et al., 2014). We and others previously reported PknA mediated regulation of morphological changes associated with bacterial cell division (Chaba et al., 2002; Kang et al., 2005; Thakur and Chakraborti, 2006, 2008). Recently, these kinases were implicated in phosphorylation of mycobacterial proteasome (Anandan et al., 2014). Thus, both these kinases play a pivotal role in governing mycobacterial physiology.

In this article, we report the ability of both PknA and PknB to transphosphorylate mDAC *in vitro*. Consistent with this observation, we found that co-expression of mDAC and PknA or PknB in *E. coli* yielded phosphorylated deacetylase protein. However, level of PknA mediated phosphorylation of mDAC was significantly high compared to that of the PknB. The phosphorylated protein exhibited decreased enzyme activity compared to mDAC. Mass spectrometric analysis of the trypsin digested fragments of the phosphorylated mDAC identified phosphorylation of protein at different serine and threonine residues. Among them, mutational analysis established the leading contribution of Thr-214 in transphosphorylation of mDAC, its enzymatic activity and its functionality as well. Furthermore, Thr-214 is conserved among different mDAC orthologs analyzed and therefore, argued the existence of cross-talk between two independent post-translational events, i.e., phosphorylation mediated control of deacetylase activity in mycobacteria.

## Materials and Methods

### Constructs, Expression, and Purification of Recombinant Proteins

Genomic DNA isolated from *M. tuberculosis* H37Ra (avirulent strain) was utilized for PCR amplification of m-*dac* (Rv1151c) and *pknB* (Rv0014c). These genes exhibited 100% identity in their nucleotide sequences compared to that of *M. tuberculosis* H37Rv (virulent strain). For this, primers (m-*dac*: 5′ AATTGGATCCCATATGCGAGTAGCGGTGC 3′ and 5′ AATTAAGCTTCTATTTCAGGAGGGCGGGCA 3′; *pknB*: 5′ AATTAGGATCCCATATG ACCACCCCTCCC 3′ and 5′ ACTGCAAGCTTCTACTGGCCGAACCT 3′) were designed incorporating restriction sites. All PCR reactions were carried out using Herculase fusion DNA polymerase as per standardized protocol (denaturation: 5 min at 95°C; reaction: 1 min at 95°C, 0.5 min at 58.7°C, 0.5 min at 72°C for 29 cycles; final extension: 10 min at 72°C). The PCR amplicons following restriction digestion(s) and subsequent purification were ligated at corresponding sites of vectors (NdeI/HindIII of pET28c/pVV2 or BamHI/HindIII of pMAL-c2X/p19kproHA) to obtain pET-mDAC or its variants or pVV-mDAC/-mDAC-T214A or pMAL-mDAC or pMAL-PknB or p19kproHA-PknB constructs. For use in some experiments, m-*dac* was PCR amplified using primers incorporating BamHI/HindIII sites and cloned in corresponding sites (in MCS1) of pETDuet-1 having *pknA* at NdeI/KpnI sites (in MCS2). Besides these, pMAL-PknA, p19kpro-PknA (Rv0015c either in pMAL or p19kpro), and pMAL-PPP (Rv0018c in pMAL) used in this study were described elsewhere (Thakur and Chakraborti, 2006, 2008).

To generate point mutants of mDAC (S38A/T39A/S179A/T197A/S212A/T214A/T214E/S222A) or PPP (G117D), two external and two internal primers (incorporating the desired mutations) were designed. This was followed by two sets of primary PCR reactions using pET-mDAC/pMAL-PPP as the template and single set of secondary PCR (using 1:1 mixture of primary PCR reaction products as template) by overlap extension method (Higuchi et al., 1988). All constructs were individually transformed in *E. coli* strains DH5α/TB1 to build up plasmid DNA and also in BL21(DE3) cells (except those in pMAL which were in TB1/DH5α cells only) for the expression as well as purification of recombinant proteins. Authenticity of all gene sequences was ensured through sequencing using an automated DNA sequencer (Applied Biosystems).

Overnight cultures (14 h at 37°C) of *E. coli* BL21(DE3) or TB1 cells harboring different constructs were re-inoculated (1% inoculum) in fresh media (LB broth) supplemented with appropriate antibiotics (100 μg/ml ampicillin for constructs in pMAL and 50 μg/ml kanamycin for pET28c vectors), grown till OD_600_ of 0.6 and then induced with 0.4 mM IPTG (5 h at 23–25°C for His-tagged constructs or 3 h at 37°C for MBP-tagged constructs). Cells were harvested, re-suspended in lysis buffer (50 mM Tris buffer, pH 7.5 containing 150 mM NaCl for His-tagged protein or 20 mM Tris buffer, pH 7.5 containing 200 mM NaCl for MBP-tagged protein and supplemented with 1 mM phenylmethylsulfonyl fluoride, 1 μg/ml pepstatin and 1 μg/ml leupeptin) and sonicated for 10 min (amplitude: 20%, frequency: 10 s ‘on’ and 15 s ‘off’) at 4°C. For His-tagged proteins, supernatant fraction was loaded onto a Ni-NTA column, washed with 20 ml of lysis buffer containing 20 mM imidazole. The resin bound protein was then eluted in elution buffer (1 ml of lysis buffer containing 50 or 100 or 150 mM imidazole). Eluted protein fractions were pooled, diluted five times in lysis buffer and reloaded onto the column to remove contamination. Column was further washed with 20 mM imidazole and 1 M NaCl followed by elution as mentioned above (eluted protein is pooled so that final imidazole concentration was 100 mM). Imidazole from the protein preparations was usually not removed since its presence did not affect the enzyme activity. MBP-tagged protein, on the other hand, was purified using an amylose column and eluted with 10 mM maltose following manufacturer’s (New England Biolabs, USA) recommended protocol. Protein concentrations of eluted samples were estimated by Bradford method (Bradford, 1976) and stored in aliquots at –80°C until used for assays. Purified phosphorylated mDAC protein, prepared from *E. coli* strain BL21(DE3) cells harboring pETDuet-mDAC/PknA, was utilized for carrying out kinetics of deacetylase activity or mass spectrometric experiments. Culture of *M. smegmatis* strain mc^2^155, transformation of pVV-mDAC and purification of recombinant protein is described elsewhere (Thakur and Chakraborti, 2008).

### Kinase Assays

Ability of PknA/PknB to transphosphorylate mDAC was determined in an *in vitro* kinase assay described elsewhere (Av-Gay et al., 1999; Chaba et al., 2002). Briefly, PknA/PknB/PknA-K42N protein (1 μg/reaction) in 1X kinase buffer (50 mM Tris-Cl pH 7.5/50 mM NaCl containing MnCl_2_, 0.51 μM for PknA/PknA-K42N or 0.46 μM for PknB) and 2 μCi of [γ- ^32^P]-ATP (specific activity: 3000–5000 Ci/mmol; purchased from Jonaki Laboratories, Board of Radiation and Isotope Technology, Hyderabad, India) was incubated in the presence of mDAC (1.79 μM/reaction) at 25°C for 30 min (total reaction volume = 20 μl). The reaction was stopped by adding 5x SDS buffer. Samples were resolved in 12% SDS PAGE and gels were stained with Coomassie Brilliant Blue. Finally, gels were analyzed in a phosphoimaging device (Fuji Film model FLA 9000) and also exposed to Kodak X-Omat/AR film for autoradiography. The dephosphorylation activity of PPP was monitored following its (5 μM/reaction) incubation (25°C for 1 h) with samples undergone kinase reaction. Unless mentioned otherwise, all experiments were carried out for at least three times.

### Deacetylase Activity

The deacetylase activity of mDAC was determined using ‘*Color de Lys* assay system,’ a microtiter plate (96 well) based colorimetric assay kit of Enzo Life Sciences. Briefly, mDAC protein (2.9 μM) and acetylated ‘*Color de Lys’* substrate peptide (final concentration 500 μM) in assay buffer (50 mM Tris-Cl, pH 8.0 containing 137 mM NaCl, 2.7 mM KCl, 1 mM MgCl_2_) were incubated with varying concentrations of NAD^+^ (0–16 mM) at 37°C for 120 min (total reaction volume = 50 μl). This was followed by addition of equal volume (50 μl) of ‘*Color de Lys’* developer solution to terminate the reaction (incubation at 37°C for 10 min) and the absorbance was measured at 405 nm in a micro-plate reader (Molecular Devices Spectramax plus 384 equipped with Softmax Pro 5.2 software). The amount of product formed was calculated by subtracting the blank (all components of reaction except the NAD^+^) readings and the specific enzyme activity (μM deacetylated peptide produced/min/mg of protein) was assessed from standard curves prepared with ‘*Color de Lys’* deacetylated standard (0–200 μM). *K*_m_ and *V*_max_ values were obtained from non-linear fit plots and the molecular mass of mDAC protein was considered as 28 kDa for calculating *k*_cat_. Unless mentioned otherwise, the experiments in the present study were done three times and data is represented as mean ± SD.

### Monitoring of Bacterial Growth

*Escherichia coli* strain BW25113 (hereafter designated as wild-type) and deacetylase gene knockout strain JW1106 (*cobB* deleted variant hereafter referred as *Δdac*) were obtained from Coli Genetic Stock Center, Yale University, USA. The strains were revived in LB broth (37°C at 200 rpm) and streaked on LB (wild-type) or LB supplemented with 50 μg/ml kanamycin (*Δdac*) plates. Growth profile of these strains using nutrient rich conditions (LB medium) was monitored for 12 h following re-inoculation of overnight cultures (initial OD_600_ = 0.1–0.15). Growth of these strains in nutrient deprived conditions was assessed using acetate medium (minimal medium comprising of 47.7 mM Na_2_HPO_4_, 25 mM KH_2_PO_4_, 9.3 mM NaCl, 17.1 mM NH_4_Cl, 2.0 mM MgSO_4_, 0.1 mM CaCl_2,_ 0.03 mM thiamine, 0.036 mM FeSO_4_.7H_2_O, and supplemented with 0.2% acetate). For this, overnight cultures (LB medium) were re-inoculated in acetate medium and grown following similar conditions with extended duration of the culture (usually 37°C at 200 rpm; sometimes cultures in between were grown in stagnant condition for ∼6 h and then shaking was re-introduced). In studies monitoring the ability of mDAC to complement *E. coli* CobB function, *Δdac* strain transformed with pMAL or pMAL-mDAC or pMAL-mDAC-T214A or pMAL-mDAC-T214E was selected on LB-ampicillin (100 μg/ml) plates. One such colony was grown overnight in LB-ampicillin medium, either inoculated in acetate containing ampicillin (100 μg/ml) for growth curve or made competent by CaCl_2_ method (Cohen et al., 1972) for further transformation with p19kpro or p19kpro-PknA or p19kpro-PknA-K42N. The effect of PknA on cell growth was monitored in acetate medium containing hygromycin (200 μg/ml) and ampicillin (50 μg/ml).

### Western Blotting

Purified fusion proteins or whole cell lysates were resolved in SDS PAGE (12%) and transferred to nitrocellulose membrane (0.45 μ) at 120 V for 1 h using a mini-transblot apparatus (BioRad, USA). Following transfer, incubation with different primary antibodies (1:1000 dilution for anti-pThr, 1:3000 dilution of anti-His and 1:25,000 of HRP conjugated anti-MBP antibodies) was carried out. For secondary antibody incubation, 1:5000 dilution of HRP conjugated anti-rabbit (for anti-pThr) or anti-mouse (for anti-His) IgG was used. The blots were finally developed for the signal through Luminata forte^TM^ following the manufacturer’s (Millipore, USA) recommended protocol. Scanning of blots was carried out using Biorad-GS-800 Calibrated Densitometer equipped with Umax Magic Scan V5.2.

### Mass Spectrometry

Phosphorylated and unphosphorylated His-tagged mDAC proteins were subjected to trypsin digestion (in-solution) in 100 mM ammonium bicarbonate buffer, pH 8.5 at 37°C for 16 h. Following digestion, 0.5% formic acid (final concentration) was added to the peptides before running them on UHPLC system (1290 series, Agilent Technologies, USA) with C8 column coupled to 6550 iFunnel QTOF LC-MS (Agilent Technologies). Electron spray ionization (ESI) was operated in positive ion mode. The instrument acquired peptide ions over the m/z range of 100–3200 with an MS and MS/MS acquisitions rates of 2 and 4 spectra/second, respectively. Peptide sequences were identified utilizing ProteinPilot^TM^ software^1^.

### CD Spectroscopy

CD spectra of wild-type mDAC and different mutant (mDAC-T214A and mDAC-T214E) proteins were compared using a Jasco J-815 spectropolarimeter. Protein solutions (0.2–0.35 mg/ml) employing a cell with path length of 0.1 cm at 25°C was used for measurements in the far ultraviolet region (250–198 nm). Each spectrum reported is an average of 3 scans and the mean residue ellipticity (𝜃) was calculated considering 110 Da as the mean of amino acid residue molecular mass.

### Bioinformatic Analyses

Multiple sequence alignment was carried out by Muscle algorithm (Edgar, 2004) using Mega 6.0 software (Tamura et al., 2013) or by Clustal Omega program (Sievers et al., 2011). PSIPRED server was used for prediction of secondary structure of the mDAC protein (McGuffin et al., 2000). Tertiary structure of mDAC was modeled using I-TASSER server (Zhang, 2008; Roy et al., 2010; Yang and Zhang, 2015; Yang et al., 2015) based on deacetylase from *Archaeoglobus fulgidus* (PDB ID: 1ICI) which has been complexed with NAD^+^ (Min et al., 2001).

## Results

### Eukaryotic-Type Ser/Thr Kinase Mediated Phosphorylation of mDAC

**FIGURE 1:**
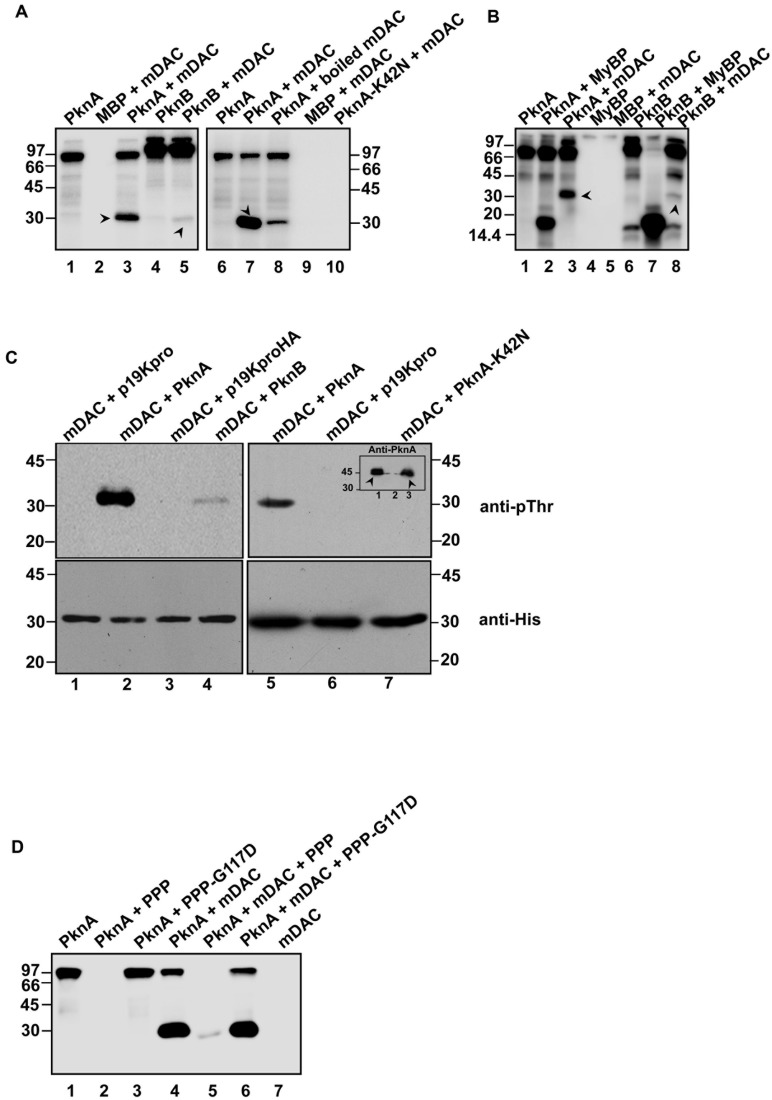
**Phosphorylation of mDAC. (A)** PknA or PknB transphosphorylates mDAC *in vitro*. MBP-tagged kinases (PknA or PknB or PknA-K42N) purified from *Escherichia coli* were incubated with [γ- ^32^P]-ATP in presence or absence of mDAC. The reactions were stopped by adding 5x SDS gel loading dye and resolved on 12% SDS-PAGE gel. The gel was further processed and analyzed utilizing a phosphoimaging device. **(B)** Comparison of the efficiency of mDAC transphosphorylation by PknA and PknB. Both the kinases were incubated with equal amounts of myelin basic protein or mDAC (1 μg) in kinase assay. Arrow heads denote His-tagged mDAC protein. **(C)** *In vivo* phosphorylation of His-tagged mDAC by PknA or PknB. Following co-transformation of pET-mDAC and p19kpro/p19kproHA or p19kpro-PknA/p19kpro-PknA-K42N/p19kproHA-PknB in BL21(DE3) cells, supernatant fraction of lysate was purified through Ni-NTA column. The purified protein samples were immunoblotted using anti-pThr (upper) and anti-His (lower) antibodies. Inset: Cell lysates of the cultures co-expressing pET-mDAC with p19kpro-PknA (lane 1) or p19kpro (lane 2) or p19kpro-PknA-K42N (lane 3) were probed with anti-PknA antibody. Arrow heads denote the expressed PknA or PknA-K42N. **(D)** Phosphorylation of mDAC is reversed by PPP. Dephosphorylation of phosphorylated mDAC was assessed following incubation with PPP/PPP-G117D using the protocol mentioned earlier. The numbers denote molecular mass markers in kDa. Notations used: PPP, phosphoprotein phosphatase; HA, hemagglutinin tag; MyBP, myelin basic protein.

Sirtuin activity in eukaryotes is reported to be influenced by the phosphorylation state of the protein (Gerhart-Hines et al., 2011). To gain an insight into phosphorylation of mycobacterial sirtuin by PknA or PknB, kinase assays were performed with or without recombinant mDAC protein. As shown in **Figure 1A** (left), both PknA and PknB were able to transphosphorylate mDAC. However, magnitude of PknB mediated phosphorylation, as adjudged by scanning the band intensity, was considerably low compared to that of PknA (compare lanes 3 and 5). To ensure phosphorylation is not an experimental artifact, we used boiled mDAC in the incubation mixture with kinase, which led to a significant decrease in the level of its transphosphorylation (**Figure 1A**, compare lanes 3 and 7 as opposed to lane 8). The use of PknA-K42N protein (a kinase-dead mutant of PknA) in the reaction mixture did not show any phosphorylation of mDAC (**Figure 1A**, lane 10). Furthermore, we used myelin basic protein, a common substrate for both PknA and PknB, as a positive control to monitor its transphosphorylation by these kinases. As expected, myelin basic protein was efficiently phosphorylated by both the kinases, which ensured that the difference in the level of phosphorylation of mDAC by PknA or PknB was not because of any discrepancy in their transphosphorylation abilities (**Figure 1B**).

To elucidate, whether mDAC is phosphorylated by PknA or PknB *in vivo*, we co-expressed p19kpro-PknA or p19kproHA-PknB and pET-mDAC in *E. coli* strain BL21(DE3) and the transformants were selected over hygromycin (200 μg/ml) and kanamycin (25 μg/ml). Cell extracts from such transformants expressing both the proteins (PknA+mDAC or PknB+mDAC) were purified through Ni-NTA and assessed for recognition by anti-pThr or anti-His antibodies in western blotting. As depicted in **Figure 1C** (upper), anti-pThr antibody recognized mDAC protein when co-expressed with kinases, such as PknA (lanes 2 and 5) and PknB (lane 4). PknA/PknB mediated phosphorylation of mDAC was further evident by the fact that anti-pThr antibody did not recognize mDAC, when co-expressed with either vector alone (p19kpro/p19kproHA) or kinase-dead variant (PknA-K42N) of PknA (**Figure 1C**, upper; compare lanes 2 and 5 with 6 and 7). However, the intensity of phosphorylated mDAC band was significantly increased when co-expressed with PknA but not with PknB (**Figure 1C**, upper left; compare lanes 2 and 4). The loading of samples was ensured in western blotting with anti-His antibody (**Figure 1C**, lower). Similarly, expression of PknA or PknA-K42N was ascertained by western blotting of lysate using anti-PknA antibody (inset, **Figure 1C**, right upper).

Since the only Ser/Thr phosphatase present in the *M. tuberculosis* genome is known to reverse auto- or transphosphorylation activity of mycobacterial kinases (Chopra et al., 2003), we monitored dephosphorylation of mDAC phosphorylated using PknA (**Figure 1D**, lane 5). The reversible phosphorylating ability of PPP was further supported by the action of phosphatase-dead mutant PPP-G117D, which did not remove phosphate from phosphorylated mDAC (**Figure 1D**, lane 6).

**FIGURE 2:**
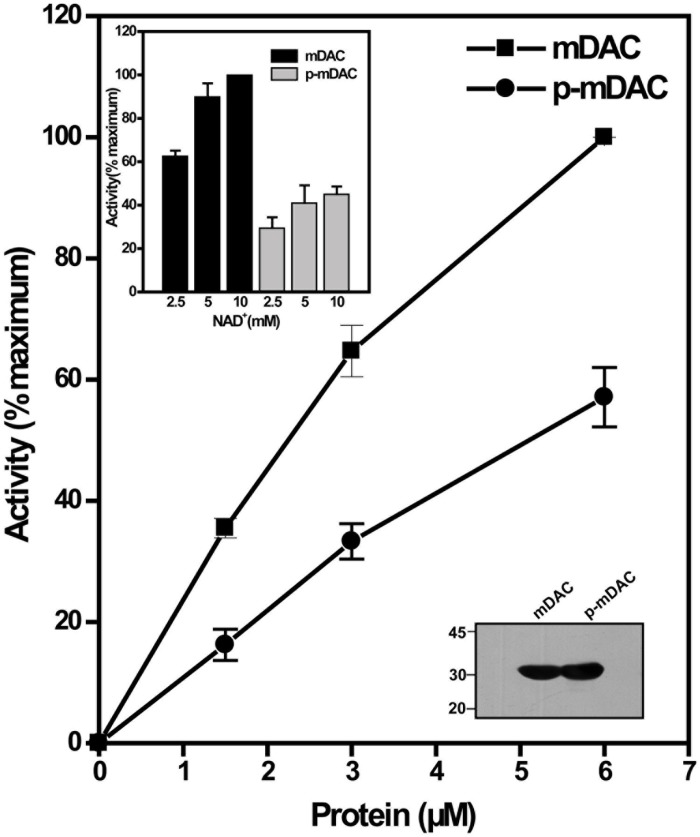
**Deacetylation activity of phosphorylated mDAC.** mDAC proteins (unphosphorylated and phosphorylated) were purified from BL21(DE3) cells using Ni-NTA based affinity columns. Commercially available ‘*Color de Lys* assay system’ was used to determine the deacetylase activity under increasing concentrations of protein (1.45–5.80 μM). Results are represented as percentage maxima (100% = 1.13 ± 0.12 μM/min with 5.8 μM protein, *n* = 4). Inset (Upper) depicts the enzyme activity of mDAC and p-mDAC in response to increasing NAD^+^ concentration (mDAC: *n* = 3; p-mDAC: *n* = 3 and 100% = 193.4 ± 15.4 μM/min/mg in 50 μl of reaction). Inset (Lower) shows western blot with anti-His antibody of unphosphorylated and phosphorylated mDAC (4 μg or 2.9 μM/lane) used for activity assays.

To monitor the effect of phosphorylation on the deacetylase activity, phosphorylated His-tagged protein (p-mDAC) was prepared from cultures of *E. coli* strain BL21(DE3) transformed with pETDuet-mDAC/PknA. Assessment of the enzymatic activity as the function of protein concentrations indicated that about twofold more p-mDAC enzyme was necessary to obtain activity at equivalent range exhibited by mDAC (**Figure 2**). In accordance with this observation, even at substrate saturated conditions (10 mM NAD^+^ with 2.9 μM protein), p-mDAC exhibited ∼45% activity compared to unphosphorylated mDAC (inset, **Figure 2**). Thus, eukaryotic-type Ser/Thr kinase, particularly PknA mediated transphosphorylation of mDAC modulated its catalytic activity.

### Thr-214 Is Predominantly Phosphorylated in mDAC

**FIGURE 3:**
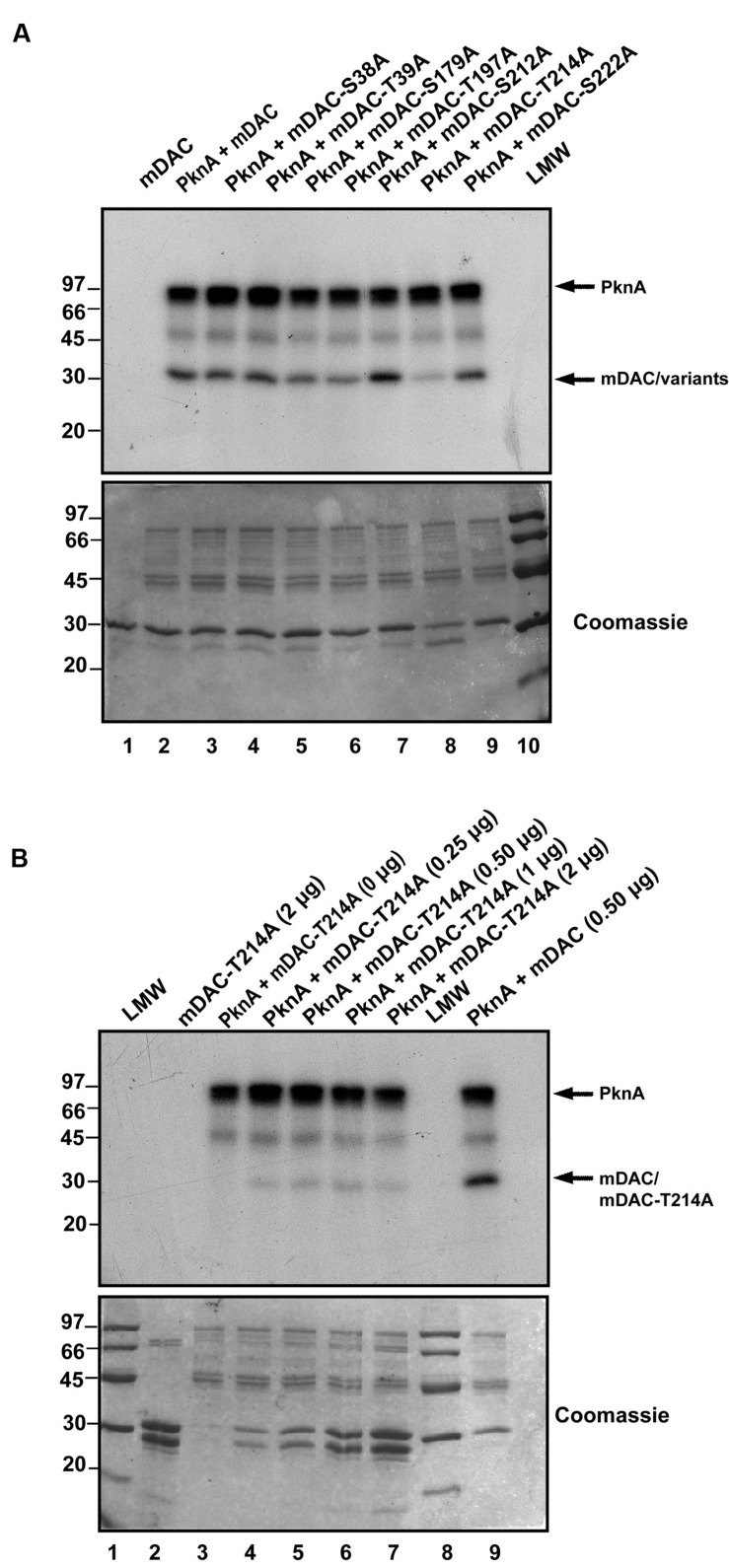
**Identification of major phosphosites in mDAC. (A)** Effect of mutations on PknA mediated phosphorylation of mDAC or its variants. Kinase assay with PknA (1 μg or 0.56 μM/assay) and mDAC or its variants (1 μg/assay) was carried out as described in Section ‘Materials and Methods.’ This is a representation of three different experiments from two independent preparations. **(B)** Phosphorylation of mDAC-T214A variant remains unaltered at increasing concentrations. Transphosphorylation of mDAC-T214A (0–2 μg) was assessed in presence of PknA (1 μg) using *in vitro* kinase assay. Wild-type mDAC (500 ng) incubated with PknA was taken as a control. Samples were resolved in 12% SDS-PAGE gel. Gels in **(A)** and **(B)** represents autoradiographs (Upper) while Coomassie stained radioactive gels served as loading controls (Lower). Notations used: LMW, low molecular weight marker.

Phosphorylated His-tagged *M. tuberculosis* sirtuin (p-mDAC) was prepared from cultures of *E. coli* strain BL21(DE3) transformed with pETDuet-mDAC/PknA. Mass spectrometric analysis (LC-MS/MS) of p-mDAC protein after trypsin digestion yielded peptides (**Table 1**; also see Supplementary Figure S1), which phosphorylated at several serine and threonine residues (Ser-38, Thr-39, Ser-179, Thr-197, Ser-212, Thr-214, and Ser-222). To explicate their contribution, we generated seven point mutants replacing Ser or Thr with Ala (S38A/T39A/S179A/T197A/S212A/T214A/S222A) and assessed PknA mediated transphosphorylation of these proteins. Interestingly, none of the mutations resulted in complete loss of phosphorylation; however, three of them exhibited either slight (mDAC-S179A/T197A) or considerable (mDAC-T214A) reduction in PknA mediated transphosphorylation abilities (**Figure 3A**). In fact, compared to mDAC, mDAC-T214A variant exhibited decrease in PknA-mediated transphosphorylation ability in a dose-dependent manner (**Figure 3B**); indicating leading role of Thr-214 in PknA mediated transphosphorylation of mDAC.

**Table 1:** Phosphosites in phosphorylated mDAC.

Peptide	Sequence	Phosphosite(s) in p-mDAC
31–44	FDPYELSS^∗^TQGWLR	Ser-38
31–44	FDPYELSST^∗^QGWLR	Thr-39
162–195	SAVEATGSADVMVVVGTS^∗^AIVYPAAGLPDLALAR	Ser-179
196–218	GT^∗^AVIEVNPEPTPLSGSATISIR	Thr-197
196–218	GTAVIEVNPEPTPLSGS^∗^ATISIR	Ser-212
196–218	GTAVIEVNPEPTPLSGSAT^∗^ISIR	Thr-214
219–231	ESAS^∗^QALPGLLER	Ser-222

^∗^*Phosphorylated amino acid.*

### Phosphorylation at Thr-214 Contributes in Catalytic Activity and Functionality of mDAC

**FIGURE 4:**
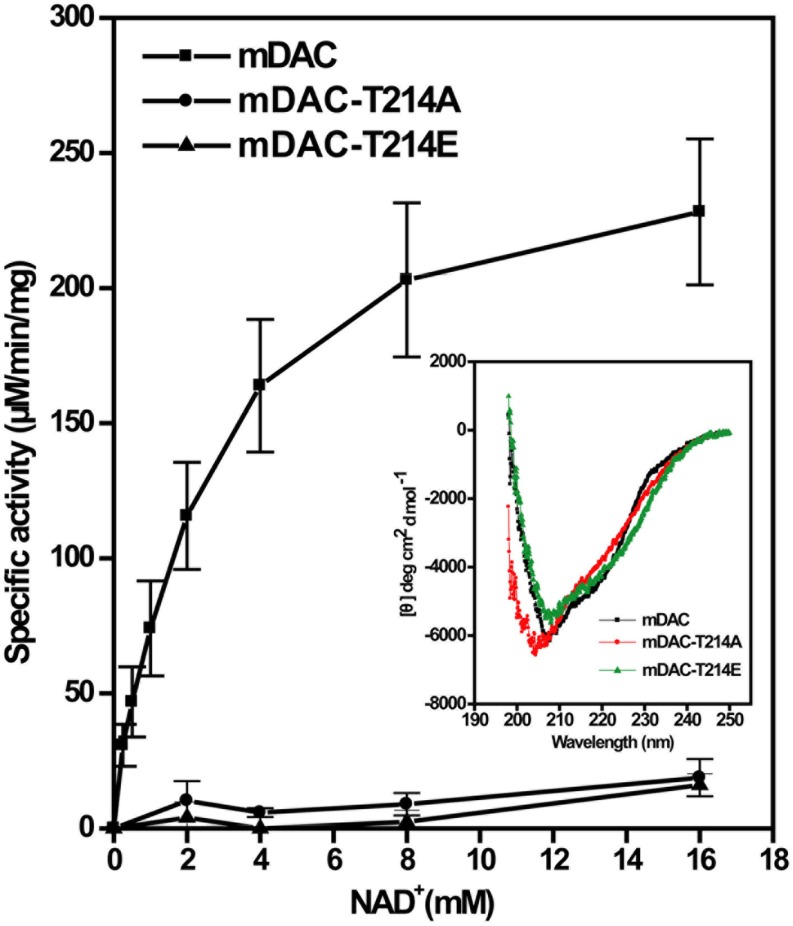
**Catalytic activity of mDAC in response to mutations at Thr-214.** Enzyme assays (4 μg or 2.9 μM protein/assay) were performed with wild-type and mDAC variants (mDAC-T214A and mDAC-T214E) as mentioned in the text in the presence of increasing concentrations of NAD^+^ (0–16 mM). Inset represents the Mean Residue Ellipticity (𝜃) identified by CD analyses for the proteins used in the assay. Microcal origin 5.0 software was used to plot ellipticity against wavelength.

Phosphorylation often provides negative charge to a protein, thereby modulating its activity (Wagner et al., 2004). To gain insight into this aspect, in addition to mDAC-T214A we generated a mutant replacing Thr-214 with a phosphomimic residue Glu. This was followed by assessment of catalytic activities of mDAC and mutants (mDAC-T214A and mDAC-T214E) as the function of increasing substrate concentrations. Kinetic analysis of deacetylase activity of mDAC protein following its incubation with increasing concentrations of NAD^+^ (0–16 mM) exhibited typical Michaelis–Menten curve (**Figure 4**) with *K*_m_ and *k*_cat_/*K*_m_ values of 2.5 ± 0.6 mM and 25.3 ± 0.6 mM^–1^.s^–1^, respectively. Contrastingly, both the mutant proteins (mDAC-T214A and mDAC-T214E) hardly showed any activity (**Figure 4**) so that their catalytic parameters could be detected. CD analysis of these mutant proteins compared to the wild-type did not depict any significant alteration in the secondary structures (inset, **Figure 4**). Since both mDAC-T214A and mDAC-T214E proteins behaved similarly (**Figure 4**), it is indicative of the contribution of phosphorylation in regulating mDAC activity irrespective of the charge at Thr-214.

**FIGURE 5:**
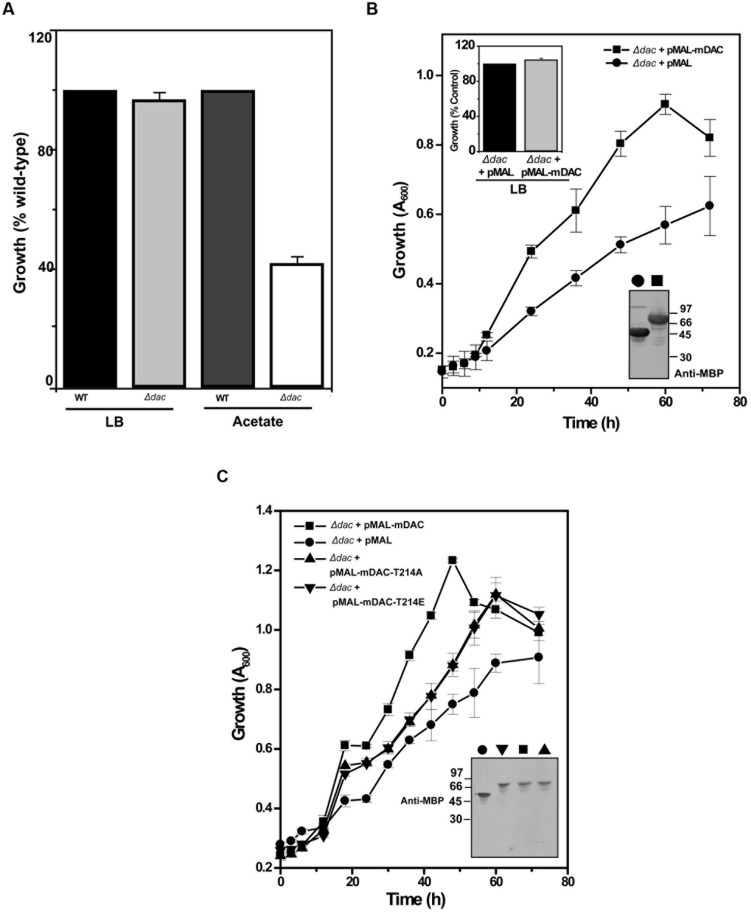
*****E. coli*** cell growth in response to mDAC phosphorylation. (A)** Effect of *cobB* gene deletion on *E. coli* growth under nutrient deprived and rich media. Growth of *Δdac* cells was expressed as percent wild-type in LB (A_600_ for wild-type at 12 h = 3.23 ± 0.04, *n* = 3) and acetate (A_600_ for wild-type at 24 h = 1.17 ± 0.004, *n* = 3) media. **(B)** Complementation of CobB functions by mDAC in *E. coli*. Growth profile of *Δdac* cells harboring pMAL (vector) or pMAL-mDAC was monitored in acetate medium for the indicated time period. Inset: Growth of *Δdac* cells harboring pMAL or pMAL-mDAC in LB medium at 12 h (Upper; A_600_ for *Δdac* cells harboring pMAL = 2.70 ± 0.28, *n* = 4) and western blot showing their expression using anti-MBP antibody (Lower; symbols in main figure were used to denote same sample). **(C)** T214E leads to attenuated growth of *E. coli* in acetate media. *E. coli Δdac* cells were complemented with pMAL or pMAL-mDAC or pMAL-T214E and growth of these transformants was assessed for indicated time periods. Inset depicts western blotting of cell lysates of different samples using anti-MBP antibody. Of note, symbols in main figure were used to denote same sample in western blots. Numbers in western blots indicate molecular mass in kDa.

NAD^+^-dependent deacetylases are fairly conserved among bacteria and are involved in regulating carbon metabolism (Schwer and Verdin, 2008; Wang et al., 2010). It is well known that expression of deacetylase (CobB) is necessary for the growth of *E. coli* in nutrient deprived conditions (Castano-Cerezo et al., 2011). Thus, to establish the effect of mDAC on cellular growth, we utilized *E. coli* cells knocked-out for its endogenous copy of deacetylase (CobB which is a homolog for mDAC, hereafter termed as *Δdac*) in this study. In our preliminary experiments, we compared the growth of wild-type and *Δdac* in LB (nutrient rich) or in acetate (nutrient deprived) medium. As anticipated, there was no difference in growth pattern between *E. coli* strains in LB medium, while it was compromised in *Δdac* in nutrient deprived conditions (**Figure 5A**; also see Supplementary Figure S2). Furthermore, to assess whether CobB function could be complemented by mDAC, we transformed pMAL-mDAC in *Δdac* strain and monitored its growth in LB as well as acetate media. Since there was leaky expression of mDAC, no IPTG was used to induce expression. Interestingly, the complemented strain (*Δdac* strain transformed with pMAL-mDAC) exhibited enhanced growth in acetate medium compared to that of the knock out variant transformed with vector alone (**Figure 5B**; also see Supplementary Figure S2). As expected, the difference in growth was not observed in LB medium (upper inset, **Figure 5B**). The expression of mDAC in cell lysates was authenticated using anti-MBP antibody (lower inset, **Figure 5B**). We further transformed pMAL-mDAC-T214A or pMAL-mDAC-T214E in *E. coli Δdac* strain and monitored their growth in acetate medium. The growth in acetate medium of both the mutants (mDAC-T214A and mDAC-T214E), as assessed by monitoring OD_600_, were nearly identical and they exhibited an intermediate growth profile between the *cobB* knock out strain (*Δdac*) either transformed with vector or complemented with mDAC (**Figure 5C**). The expression of different constructs (pMAL-mDAC or pMAL-mDAC-T214A/T214E or vector) in cell lysates was confirmed in western blotting using anti-MBP antibody (inset, **Figure 5C**). Thus, our findings insinuate the role of PknA mediated phosphorylation of mDAC at Thr-214 in regulating its functionality.

**FIGURE 6:**
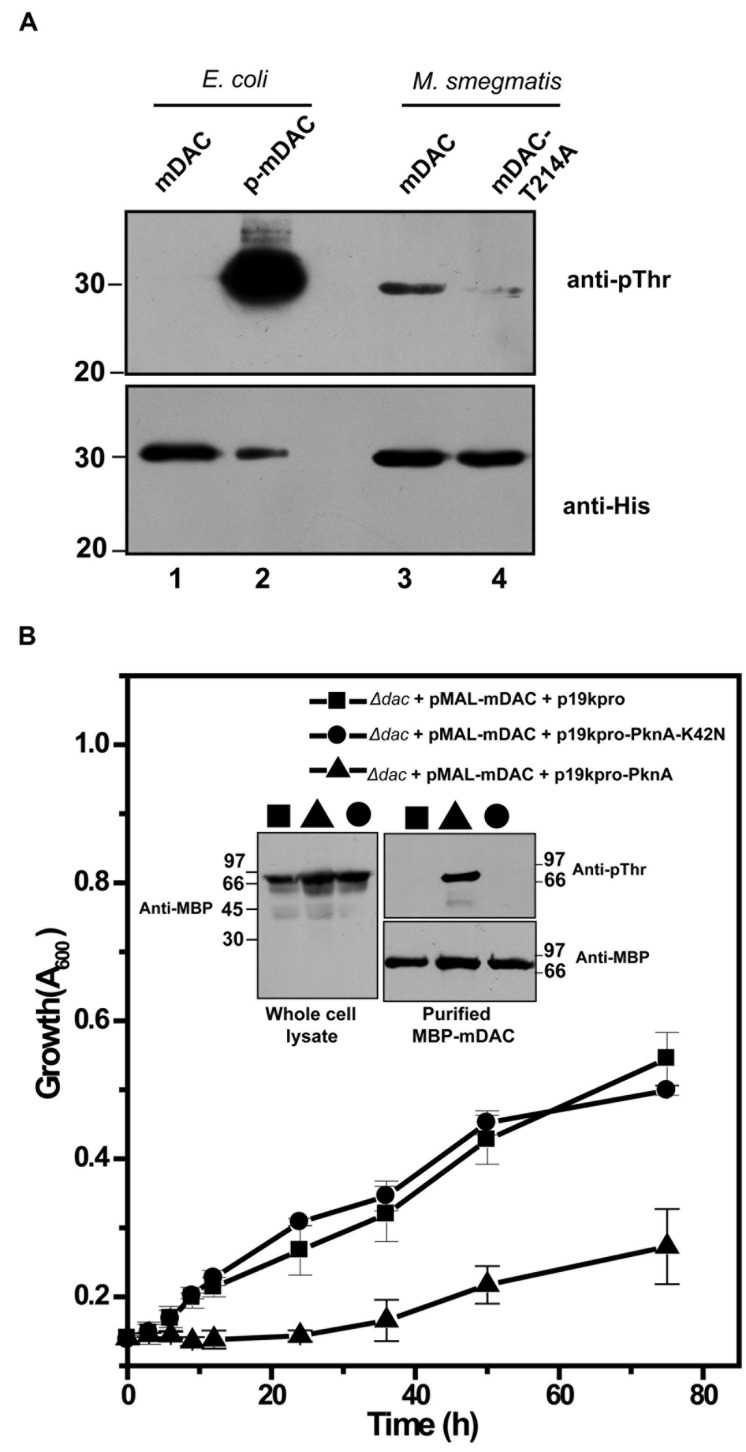
**(A)** Phosphorylation of mDAC within *M. smegmatis*. *M. smegmatis* strain mc^2^155 harboring pVV-mDAC or pVV-mDAC-T214A was cultured in LB medium (induced with H_2_O_2_ and grown in presence of Tween 80). mDAC or mDAC-T214A purified from these cultures (1 μg protein) were subjected to western blotting with anti-pThr (Upper) or anti-His (Lower) antibodies. mDAC (1 μg) and p-mDAC (500 ng) purified from *E. coli* strain BL21(DE3) were used as internal controls for the experiment. **(B)** PknA mediated phosphorylation of mDAC affects *E. coli* growth in acetate media. mDAC complemented *E. coli Δdac* cells were further co-transformed with p19kpro/p19kpro-PknA/p19kpro-PknA-K42N and growth of these transformants were assessed for indicated time periods. Inset: Western blotting of cell lysates of different samples using anti-MBP antibody (Left). Same lysates purified through amylose resin were probed with anti-pThr (Top right) and anti-MBP (Bottom right) antibodies in western blot.

The presence of homologs of *M. tuberculosis* eukaryotic-type Ser/Thr kinases, in *M. smegmatis* is well known (Thakur and Chakraborti, 2008; Prisic and Husson, 2014). We further generated pVV-mDAC or pVV-mDAC-T214A constructs and expressed them in non-pathogenic saprophyte *M. smegmatis* strain mc^2^155 under the control of an inducible promoter. We compared phosphorylation status of mDAC and mDAC-T214A proteins in western blotting with anti-pThr or anti-His antibody following purification through Ni-NTA column. While mDAC was recognized by anti-pThr antibody, there was hardly any signal for mDAC-T214A (**Figure 6A**, upper; compare lanes 3 and 4). The blot developed using anti-His antibody ruled out any discrepancy in amount of sample loading (**Figure 6A**, lower; compare lanes 3 and 4). The mDAC (phosphorylated and unphosphorylated) protein purified from *E. coli* strain BL21(DE3) was used as internal controls for the experiment to rule out any possibility of non-specificity of anti-pThr antibody. Collectively, these data strongly argue in favor of endogenous eukaryotic-type Ser/Thr kinase (like PknA) mediated phosphorylation of mDAC, when over-expressed in *M. smegmatis.*

To elucidate *in vivo* functional interaction between eukaryotic-type Ser/Thr kinase like PknA and *M. tuberculosis* sirtuin (mDAC), we further utilized *E. coli* based expression system since it does not possess such endogenous kinases or phosphatase. For this, p19kpro (vector) or p19kpro-PknA (kinase) or p19kpro-PknA-K42N (kinase-dead mutant) was transformed in mDAC complemented *Δdac* strain and grown in nutrient deprived condition (acetate medium). Interestingly, growth was affected in the presence of PknA compared to that of the vector control (p19kpro). This effect was kinase specific since there was no *E. coli* growth inhibition in the presence of the kinase-dead variant, PknA-K42N (**Figure 6B**). The expression of mDAC in cell lysates (inset, **Figure 6B**) and phosphorylation of protein(s) from cultures co-expressing p19kpro-PknA and MBP-mDAC following purification through amylose resin (inset, **Figure 6B**) was confirmed by western blotting using appropriate (anti-MBP/anti-pThr) antibodies. Thus, our findings strongly suggest that PknA mediated phosphorylation of mDAC affected functionality of this protein.

### Thr-214 Is Conserved among Mycobacterial Sirtuin Orthologs

**FIGURE 7:**
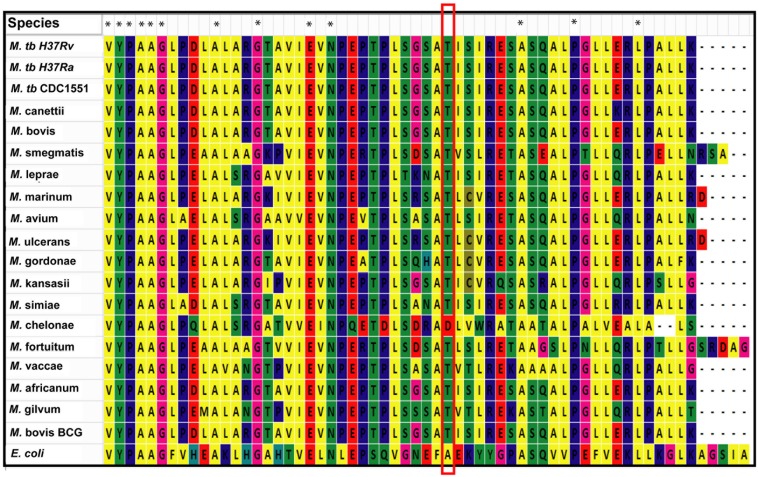
**Thr-214 is conserved in mDAC orthologs.** Multiple sequence alignment utilizing NAD^+^-dependent deacetylases (sequences procured from MycoRRdb database and NCBI) from different mycobacterial species was performed by Muscle algorithm using Mega 6.0 software. Figure represents the C-termini of the deacetylase sequences. Conserved threonine residue is enclosed in red rectangular box. CobB from *E. coli* was used as an out-group for the analysis. Notations used: *M., Mycobacterium*; *E*., *Escherichia*. Asterisk (^∗^) indicates identical residue.

We used MycoRRdb database (Midha et al., 2012), NCBI BLAST and manual curated search to obtain the NAD^+^-dependent deacetylases from different mycobacterial species. Protein sequences for mycobacterial deacetylases along with those belonging to different taxa were procured using NCBI^2^. Multiple sequence alignment by Muscle and Clustal omega revealed that mDAC is very much conserved in almost all mycobacterial species (**Figure 7**) but far distinct when compared with deacetylase sequences from other taxa representing its unique phylogenetic distribution. Of note, the deacetylase sequence from *E. coli* was taken as an out-group for the analysis. Furthermore, when closely examined Thr-214 remains conserved in all mycobacterial deacetylases except for *M. chelonae* which bears Asp at this position (**Figure 7**). Thus, it seems that phosphorylation at this threonine residue is conserved in all mycobacterial deacetylases.

## Discussion

Sirtuins in eukaryotes are involved in variety of diverse cellular processes that includes control of life span extension (Masoro, 2004), transcriptional regulation (Yeung et al., 2004), DNA repair (Li et al., 2011), stress resistance, and metabolism (Wang et al., 2010; Ma and Wood, 2011). In prokaryotes, their involvement in controlling enzymes associated with central carbon metabolism has already been recognized (Wang et al., 2010). Accordingly, we focussed on phosphorylation/de-phosphorylation mediated control of the only sirtuin (mDAC) present in *M. tuberculosis* genome that displays NAD^+^-dependent deacetylase activity. Since regulatory interplay between different signaling events is critical for adaptation of any micro-organism in a particular environment, such cross-talk is definitely important to elucidate. In fact, enzymes associated with these events are considered as vital targets for screening or designing of new anti-mycobacterials (Rhee et al., 2011).

We observed that mDAC was transphosphorylated by PknA, a representative of mycobacterial eukaryotic-type Ser/Thr kinases (**Figure 1**). Our results also indicated that co-expression of PknA and mDAC in *E. coli* yielded p-mDAC protein, which was recognized by anti-pThr antibody in western blotting. The phosphorylated protein (p-mDAC) exhibited decreased deacetylase activity compared to its unphosphorylated counterpart (**Figures 1** and **2**). Mass spectrometric studies with p-mDAC identified four serines (amino acids 38, 179, 212, and 222) and three threonines (amino acids 39, 197, and 214) as phosphorylating residues (**Table 1**). Mutation (one at a time) of these residues to Ala followed by kinase assay with these proteins in the presence of PknA, however, indicated the role of Ser-179, Thr-197, and Thr-214 in PknA mediated phosphorylation of mDAC (**Figure 3**). Amongst these residues, we noticed major contribution of Thr-214 for transphosphorylation of mDAC (**Figure 3**).

To evaluate the effect of phosphorylation on the catalytic activity of the protein, we compared mDAC along with its phospho-ablated (mDAC-T214A) or phosphomimic (mDAC-T214E) variants. To our surprise, both the mutant proteins displayed hardly any enzymatic activities indicating direct role of phosphorylation in the process (**Figure 4**). To establish functional implications of this finding, we opted for a phenotypic assay, where expression of NAD^+^-dependent deacetylase, CobB is necessary for optimal growth of *E. coli* in nutrient deprived acetate medium (Castano-Cerezo et al., 2011). Furthermore, mDAC was able to complement CobB function (since mDAC shares ∼52% sequence homology with CobB and most NAD^+^-dependent deacetylases are functionally similar) in its knock out *Δdac* strain (**Figure 4B**). Unlike mDAC, neither its phospho-ablated nor phosphomimic variant complemented growth phenotype, indicating the role of phosphorylation at Thr-214 in controlling the enzymatic activity as well as functionality of mDAC (**Figures 4** and **5**).

*Mycobacterium smegmatis*, where endogenous eukaryotic-type Ser/Thr kinases, like PknA and PknB are present, is often used as a genetic model in identifying interacting partners for such kinases from *M. tuberculosis* (Villarino et al., 2005; Thakur and Chakraborti, 2008; Plocinski et al., 2014; Prisic and Husson, 2014). We further examined phosphorylation status of mDAC or mDAC-T214A upon overexpression in *M. smegmatis*. The mDAC protein was recognized by anti-pThr antibody in western blotting indicating its *in vivo trans*-phosphorylating ability by endogenous kinases of *M. smegmatis*. However, the extent of phosphorylation, as adjudged by scanning the band intensity of the protein, was drastically low in mDAC-T214A compared to that of the mDAC (**Figure 6A**). These results, however, do not pin point *in vivo* functional interaction of a specific kinase in the process. Given the fact that there are several eukaryotic-type Ser/Thr kinases and a cognate phosphatase are present in all mycobacterial genomes sequenced to-date and some of them are essential in nature, it would be an arduous task to resolve this issue using *M. tuberculosis* as such. In this scenario, we again utilized mDAC complemented *E. coli Δdac* strain and transformed it with either PknA or its kinase dead variant, PknA-K42N, and monitored growth profile of the cultures in acetate medium. The outcome of this study indicated that at least PknA mediated phosphorylation of mDAC altered the growth phenotype. Thus, our results ostensibly established Thr-214 as the major phosphosite in mDAC and its phosphorylation is a eukaryotic-type Ser/Thr kinase mediated event.

Most notably, we also obtained and analyzed 1:1 sequence alignment of mDAC with each of the human sirtuins and observed NAD^+^ binding sites are fairly conserved. Interestingly, the presence of a charged residue (D/E/R) corresponding to Thr-214 of mDAC is evident, except for Cys in Sirt4 and Ala in Sirt7 (Supplementary Figure S3). In fact, sequence alignment of mDAC with gram positive bacterial deacetylases also showed the presence of charged residue matching Thr-214 (Supplementary Figure S3). Considering these results with mDAC activity assays (**Figures 2** and **4**), it seems that the generation of a charged environment corresponding to Thr-214 in other deacetylases is important in modulating their enzymatic activity. Nonetheless, it is distinct and unique in mycobacterial sirtuins where phosphorylation at Thr-214 *per se* is a molecular switch that governs NAD^+^-dependent protein acetylation/deacetylation trajectory. Although mechanistic detail of such a regulation is very remote to postulate in the absence of crystal structure of mDAC, phosphorylation induced conformational change of proteins are well known. The positioning of Thr-214 is in the flexible coiled region (Supplementary Figure S4). Furthermore, its predicted structure based on the same protein from thermostable *Archaeoglobus fulgidus* (46% identity and 62% homology) through I-TASSER server indicated Thr-214 to be in unstructured region (Supplementary Figure S4). Taken together with our results of enzymatic activity, it is very likely an indication of ‘action at distance’ control of the active site of mDAC by phosphorylation at Thr-214. Further studies in this direction, especially with mDAC crystal structure, would unravel such mystery.

Several studies indicated that primary carbon source for *M. tuberculosis* is fatty acids/cholesterol in nutrient restricted conditions as evident when bacteria reside within host macrophages (Pandey and Sassetti, 2008; Griffin et al., 2012). The degradation of fatty acids/cholesterol is mediated through fatty acyl-CoA synthetase family of proteins, which is regulated by reversible acetylation generating acetyl-CoA and propionyl-CoA (Nambi et al., 2013). In this scenario, it is tempting to speculate that phosphorylated mDAC being compromised in its ability to deacetylate the participatory enzyme(s) would be affecting the turnover of acetyl-CoA and propionyl-CoA. Since high concentrations of propionyl-CoA could be toxic for the bacterium (Upton and McKinney, 2007), it would very likely be involved in reducing toxicity caused by building up of propionyl-CoA by promoting acetylation of acetyl-CoA synthetase and/or reducing deacetylase activity of mDAC. Therefore, to provide such a rapid control of the situation, phosphorylation mediated regulation of mDAC activity seems to be the need for the bacterium for its survival. Although mammalian SIRT1 activity is known to be governed by phosphorylation (Gerhart-Hines et al., 2011), in bacteria this is the first report. Thus, it is entirely plausible that phosphorylation is a common regulatory mechanism recruited by nature to fine tune sirtuin activity in both prokaryotes and eukaryotes.

## Conclusion

Our results established that eukaryotic-type Ser/Thr kinase mediated phosphorylation of mycobacterial sirtuin modulates its enzymatic activity as well as its functionality. Furthermore, we for the first time provide evidence for cross-talk between two distinct post-translational events, phosphorylation and deacetylation, thereby regulating bacterial signaling. Further work in this direction is necessary to unravel the mechanistic details.

## Author Contributions

PC and GY conceived the idea; planned experiments, analyzed the results and wrote the manuscript; GY, SR, and NM carried out experiments.

## Conflict of Interest Statement

The authors declare that the research was conducted in the absence of any commercial or financial relationships that could be construed as a potential conflict of interest.

## Footnotes

1 http://www.absciex.com/products/software/proteinpilot-software

2 http://ncbi.nlm.nih.gov

## Abbreviations

anti-His: anti-histidine antibody
anti-pThr: anti-phosphothreonine antibody
anti-MBP: anti-maltose binding protein antibody
CD: circular dichroism
*Δdac*: *E. coli* strain JW1106 deleted with NAD^+^-dependent deacetylase
His-tag: 6X histidine tag
LC-MS/MS: liquid chromatography coupled tandem mass spectrometry
mDAC: *M. tuberculosis* NAD^+^-dependent deacetylase
MBP: maltose binding protein
NAD^+^: nicotinamide adenosine dinucleotide
p-mDAC: phosphorylated *M. tuberculosis* NAD^+^-dependent deacetylase.

## References

[B1] Ahn B. H. Kim H. S. Song S. Lee I. H. Liu J. Vassilopoulos A. (2008). A role for the mitochondrial deacetylase Sirt3 in regulating energy homeostasis. *Proc. Natl. Acad. Sci. U.S.A.* 105 14447–14452. 10.1073/pnas.0803790105 18794531 PMC2567183

[B2] Anandan T. Han J. Baun H. Nyayapathy S. Brown J. T. Dial R. L. (2014). Phosphorylation regulates mycobacterial proteasome. *J. Microbiol.* 52 743–754. 10.1007/s12275-014-4416-2 25224505

[B3] Av-Gay Y. Everett M. (2000). The eukaryotic-like Ser/Thr protein kinases of *Mycobacterium tuberculosis*. *Trends Microbiol.* 8 238–244. 10.1016/S0966-842X(00)01734-0 10785641

[B4] Av-Gay Y. Jamil S. Drews S. J. (1999). Expression and characterization of the *Mycobacterium tuberculosis* serine/threonine protein kinase PknB. *Infect Immun.* 67 5676–5682. 10531215 10.1128/iai.67.11.5676-5682.1999 PMC96941

[B5] Baer C. E. Iavarone A. T. Alber T. Sassetti C. M. (2014). Biochemical and spatial coincidence in the provisional Ser/Thr protein kinase interaction network of *Mycobacterium tuberculosis*. *J. Biol. Chem.* 289 20422–20433. 10.1074/jbc.M114.559054 24928517 PMC4110253

[B6] Bradford M. M. (1976). A rapid and sensitive method for the quantitation of microgram quantities of protein utilizing the principle of protein-dye binding. *Anal. Biochem.* 72 248–254. 10.1016/0003-2697(76)90527-3 942051

[B7] Castano-Cerezo S. Bernal V. Blanco-Catala J. Iborra J. L. Canovas M. (2011). cAMP-CRP co-ordinates the expression of the protein acetylation pathway with central metabolism in *Escherichia coli*. *Mol. Microbiol.* 82 1110–1128. 10.1111/j.1365-2958.2011.07873.x 22059728

[B8] Chaba R. Raje M. Chakraborti P. K. (2002). Evidence that a eukaryotic-type serine/threonine protein kinase from *Mycobacterium tuberculosis* regulates morphological changes associated with cell division. *Eur. J. Biochem.* 269 1078–1085. 10.1046/j.1432-1033.2002.02778.x 11856348

[B9] Chopra P. Singh B. Singh R. Vohra R. Koul A. Meena L. S. (2003). Phosphoprotein phosphatase of *Mycobacterium tuberculosis* dephosphorylates serine-threonine kinases PknA and PknB. *Biochem. Biophys. Res. Commun.* 311 112–120. 10.1016/j.bbrc.2003.09.173 14575702

[B10] Cohen S. N. Chang A. C. Hsu L. (1972). Nonchromosomal antibiotic resistance in bacteria: genetic transformation of *Escherichia coli* by R-factor DNA. *Proc. Natl. Acad. Sci. U.S.A.* 69 2110–2114. 10.1073/pnas.69.8.2110 4559594 PMC426879

[B11] Cole S. T. Eiglmeier K. Parkhill J. James K. D. Thomson N. R. Wheeler P. R. (2001). Massive gene decay in the leprosy bacillus. *Nature* 409 1007–1011. 10.1038/35059006 11234002

[B12] Crosby H. A. Heiniger E. K. Harwood C. S. Escalante-Semerena J. C. (2010). Reversible N epsilon-lysine acetylation regulates the activity of acyl-CoA synthetases involved in anaerobic benzoate catabolism in *Rhodopseudomonas palustris*. *Mol. Microbiol.* 76 874–888. 10.1111/j.1365-2958.2010.07127.x 20345662 PMC2913386

[B13] Dasgupta A. Datta P. Kundu M. Basu J. (2006). The serine/threonine kinase PknB of *Mycobacterium tuberculosis* phosphorylates PBPA, a penicillin-binding protein required for cell division. *Microbiology* 152 493–504. 10.1099/mic.0.28630-0 16436437

[B14] Edgar R. C. (2004). MUSCLE: multiple sequence alignment with high accuracy and high throughput. *Nucleic Acids Res.* 32 1792–1797. 10.1093/nar/gkh340 15034147 PMC390337

[B15] Fernandez P. Saint-Joanis B. Barilone N. Jackson M. Gicquel B. Cole S. T. (2006). The Ser/Thr protein kinase PknB is essential for sustaining mycobacterial growth. *J. Bacteriol.* 188 7778–7784. 10.1128/JB.00963-06 16980473 PMC1636329

[B16] Gerhart-Hines Z. Dominy J. E. Jr. Blättler S. M. Jedrychowski M. P. Banks A. S. Lim J. H. (2011). The cAMP/PKA pathway rapidly activates SIRT1 to promote fatty acid oxidation independently of changes in NAD(+). *Mol. Cell* 44 851–863. 10.1016/j.molcel.2011.12.005 22195961 PMC3331675

[B17] Griffin J. E. Pandey A. K. Gilmore S. A. Mizrahi V. McKinney J. D. Bertozzi C. R. (2012). Cholesterol catabolism by *Mycobacterium tuberculosis* requires transcriptional and metabolic adaptations. *Chem. Biol.* 19 218–227. 10.1016/j.chembiol.2011.12.016 22365605 PMC3292763

[B18] Gu J. Deng J. Y. Li R. Wei H. Zhang Z. Zhou Y. (2009). Cloning and characterization of NAD-dependent protein deacetylase (Rv1151c) from *Mycobacterium tuberculosis*. *Biochemistry (Mosc.)* 74 743–748. 10.1134/S0006297909070062 19747094

[B19] Guarente L. (2007). Sirtuins in aging and disease. *Cold Spring Harb. Symp. Quant. Biol.* 72 483–488. 10.1101/sqb.2007.72.024 18419308

[B20] Higuchi R. Krummel B. Saiki R. K. (1988). A general method of in vitro preparation and specific mutagenesis of DNA fragments: study of protein and DNA interactions. *Nucleic Acids Res.* 16 7351–7367. 10.1093/nar/16.15.7351 3045756 PMC338413

[B21] Hipkiss A. R. (2008). Energy metabolism, altered proteins, sirtuins and ageing: converging mechanisms? *Biogerontology* 9 49–55. 10.1007/s10522-007-9110-x 17929190 PMC2174522

[B22] Kang C. M. Abbott D. W. Park S. T. Dascher C. C. Cantley L. C. Husson R. N. (2005). The Mycobacterium tuberculosis serine/threonine kinases PknA and PknB: substrate identification and regulation of cell shape. *Genes Dev.* 19 1692–1704. 10.1101/gad.1311105 15985609 PMC1176007

[B23] Khan S. Nagarajan S. N. Parikh A. Samantaray S. Singh A. Kumar D. (2010). Phosphorylation of enoyl-acyl carrier protein reductase InhA impacts mycobacterial growth and survival. *J. Biol. Chem.* 285 37860–37871. 10.1074/jbc.M110.143131 20864541 PMC2988389

[B24] Li Z. Wen J. Lin Y. Wang S. Xue P. Zhang Z. (2011). A Sir2-like protein participates in mycobacterial NHEJ. *PLoS ONE* 6:e20045 10.1371/journal.pone.0020045 PMC3102665 21637345

[B25] Liu F. Yang M. Wang X. Yang S. Gu J. Zhou J. (2014). Acetylome analysis reveals diverse functions of lysine acetylation in *Mycobacterium tuberculosis*. *Mol. Cell Proteomics* 13 3352–3366. 10.1074/mcp.M114.041962 25180227 PMC4256489

[B26] Ma Q. Wood T. K. (2011). Protein acetylation in prokaryotes increases stress resistance. *Biochem. Biophys. Res. Commun.* 410 846–851. 10.1016/j.bbrc.2011.06.076 21703240 PMC3138907

[B27] Masoro E. J. (2004). Role of sirtuin proteins in life extension by caloric restriction. *Mech. Ageing Dev.* 125 591–594. 10.1016/j.mad.2004.08.011 15491676

[B28] McGuffin L. J. Bryson K. Jones D. T. (2000). The PSIPRED protein structure prediction server. *Bioinformatics* 16 404–405. 10.1093/bioinformatics/16.4.404 10869041

[B29] Midha M. Prasad N. K. Vindal V. (2012). MycoRRdb: a database of computationally identified regulatory regions within intergenic sequences in mycobacterial genomes. *PLoS ONE* 7:e36094 10.1371/journal.pone.0036094 PMC3338573 22563442

[B30] Min J. Landry J. Sternglanz R. Xu R. M. (2001). Crystal structure of a SIR2 homolog-NAD complex. *Cell* 105 269–279. 10.1016/S0092-8674(01)00317-8 11336676

[B31] Nambi S. Gupta K. Bhattacharyya M. Ramakrishnan P. Ravikumar V. Siddiqui N. (2013). Cyclic AMP-dependent protein lysine acylation in mycobacteria regulates fatty acid and propionate metabolism. *J. Biol. Chem.* 288 14114–14124. 10.1074/jbc.M113.463992 23553634 PMC3656268

[B32] Nguyen L. Walburger A. Houben E. Koul A. Muller S. Morbitzer M. (2005). Role of protein kinase G in growth and glutamine metabolism of *Mycobacterium bovis* BCG. *J. Bacteriol.* 187 5852–5856. 10.1128/JB.187.16.5852-5856.2005 16077135 PMC1196090

[B33] Pandey A. K. Sassetti C. M. (2008). Mycobacterial persistence requires the utilization of host cholesterol. *Proc. Natl. Acad. Sci. U.S.A.* 105 4376–4380. 10.1073/pnas.0711159105 18334639 PMC2393810

[B34] Pereira S. F. Goss L. Dworkin J. (2011). Eukaryote-like serine/threonine kinases and phosphatases in bacteria. *Microbiol. Mol. Biol. Rev.* 75 192–212. 10.1128/MMBR.00042-10 21372323 PMC3063355

[B35] Plocinski P. Laubitz D. Cysewski D. Stodus K. Kowalska K. Dziembowski A. (2014). Identification of protein partners in mycobacteria using a single-step affinity purification method. *PLoS ONE* 9:e91380 10.1371/journal.pone.0091380 PMC3963859 24664103

[B36] Prisic S. Husson R. N. (2014). Mycobacterium tuberculosis Serine/Threonine Protein Kinases. *Microbiol. Spectr.* 2. 10.1128/microbiolspec.MGM2-0006-2013 PMC4242435 25429354

[B37] Rhee K. Y. de Carvalho L. P. Bryk R. Ehrt S. Marrero J. Park S. W. (2011). Central carbon metabolism in *Mycobacterium tuberculosis*: an unexpected frontier. *Trends Microbiol.* 19 307–314. 10.1016/j.tim.2011.03.008 21561773 PMC3601588

[B38] Roy A. Kucukural A. Zhang Y. (2010). I-TASSER: a unified platform for automated protein structure and function prediction. *Nat. Protoc.* 5 725–738. 10.1038/nprot.2010.5 20360767 PMC2849174

[B39] Sajid A. Arora G. Gupta M. Singhal A. Chakraborty K. Nandicoori V. K. (2011). Interaction of *Mycobacterium tuberculosis* elongation factor Tu with GTP is regulated by phosphorylation. *J. Bacteriol.* 193 5347–5358. 10.1128/JB.05469-11 21803988 PMC3187401

[B40] Sassetti C. M. Boyd D. H. Rubin E. J. (2003). Genes required for mycobacterial growth defined by high density mutagenesis. *Mol. Microbiol.* 48 77–84. 10.1046/j.1365-2958.2003.03425.x 12657046

[B41] Schwer B. Verdin E. (2008). Conserved metabolic regulatory functions of sirtuins. *Cell Metab* 7 104–112. 10.1016/j.cmet.2007.11.006 18249170

[B42] Sievers F. Wilm A. Dineen D. Gibson T. J. Karplus K. Li W. (2011). Fast, scalable generation of high-quality protein multiple sequence alignments using Clustal Omega. *Mol. Syst. Biol.* 7:539 10.1038/msb.2011.75 PMC3261699 21988835

[B43] Starai V. J. Celic I. Cole R. N. Boeke J. D. Escalante-Semerena J. C. (2002). Sir2-dependent activation of acetyl-CoA synthetase by deacetylation of active lysine. *Science* 298 2390–2392. 10.1126/science.1077650 12493915

[B44] Starai V. J. Takahashi H. Boeke J. D. Escalante-Semerena J. C. (2003). Short-chain fatty acid activation by acyl-coenzyme A synthetases requires SIR2 protein function in *Salmonella enterica* and *Saccharomyces cerevisiae*. *Genetics* 163 545–555. 12618394 10.1093/genetics/163.2.545 PMC1462443

[B45] Tamura K. Stecher G. Peterson D. Filipski A. Kumar S. (2013). MEGA6: molecular evolutionary genetics analysis version 6.0. *Mol. Biol. Evol.* 30 2725–2729. 10.1093/molbev/mst197 24132122 PMC3840312

[B46] Thakur M. Chakraborti P. K. (2006). GTPase activity of mycobacterial FtsZ is impaired due to its transphosphorylation by the eukaryotic-type Ser/Thr kinase. *PknA. J. Biol. Chem.* 281 40107–40113. 10.1074/jbc.M607216200 17068335

[B47] Thakur M. Chakraborti P. K. (2008). Ability of PknA, a mycobacterial eukaryotic-type serine/threonine kinase, to transphosphorylate MurD, a ligase involved in the process of peptidoglycan biosynthesis. *Biochem. J.* 415 27–33. 10.1042/BJ20080234 18557704

[B48] Upton A. M. McKinney J. D. (2007). Role of the methylcitrate cycle in propionate metabolism and detoxification in *Mycobacterium smegmatis*. *Microbiology* 153 3973–3982. 10.1099/mic.0.2007/011726-0 18048912

[B49] Villarino A. Duran R. Wehenkel A. Fernandez P. England P. Brodin P. (2005). Proteomic identification of *M. tuberculosis* protein kinase substrates: PknB recruits GarA, a FHA domain-containing protein, through activation loop-mediated interactions. *J. Mol. Biol.* 350 953–963. 10.1016/j.jmb.2005.05.049 15978616

[B50] Wagner L. E. Li W.-H. Joseph S. K. Yule D. I. (2004). Functional consequences of phosphomimetic mutations at key cAMP-dependent protein kinase phosphorylation sites in the type 1 inositol 1, 4, 5-trisphosphate receptor. *J. Biol. Chem.* 279 46242–46252. 10.1074/jbc.M405849200 15308649

[B51] Wang Q. Zhang Y. Yang C. Xiong H. Lin Y. Yao J. (2010). Acetylation of metabolic enzymes coordinates carbon source utilization and metabolic flux. *Science* 327 1004–1007. 10.1126/science.1179687 20167787 PMC4183141

[B52] Xie L. Wang X. Zeng J. Zhou M. Duan X. Li Q. (2015). Proteome-wide lysine acetylation profiling of the human pathogen *Mycobacterium tuberculosis*. *Int. J. Biochem. Cell Biol.* 59 193–202. 10.1016/j.biocel.2014.11.010 25456444

[B53] Yang J. Yan R. Roy A. Xu D. Poisson J. Zhang Y. (2015). The I-TASSER Suite: protein structure and function prediction. *Nat. Methods* 12 7–8. 10.1038/nmeth.3213 25549265 PMC4428668

[B54] Yang J. Zhang Y. (2015). I-TASSER server: new development for protein structure and function predictions. *Nucleic Acids Res.* 43 W174–W181. 10.1093/nar/gkv342 25883148 PMC4489253

[B55] Yeung F. Hoberg J. E. Ramsey C. S. Keller M. D. Jones D. R. Frye R. A. (2004). Modulation of NF-kappaB-dependent transcription and cell survival by the SIRT1 deacetylase. *EMBO J.* 23 2369–2380. 10.1038/sj.emboj.7600244 15152190 PMC423286

[B56] Zhang K. Zheng S. Yang J. S. Chen Y. Cheng Z. (2013). Comprehensive profiling of protein lysine acetylation in *Escherichia coli*. *J. Proteome Res.* 12 844–851. 10.1021/pr300912q 23294111

[B57] Zhang Y. (2008). I-TASSER server for protein 3D structure prediction. *BMC Bioinformatics* 9:40 10.1186/1471-2105-9-40 PMC2245901 18215316

[B58] Zhao S. Xu W. Jiang W. Yu W. Lin Y. Zhang T. (2010). Regulation of cellular metabolism by protein lysine acetylation. *Science* 327 1000–1004. 10.1126/science.1179689 20167786 PMC3232675

