# Supplementary material for: Phosphorylation Modulates Catalytic Activity of Mycobacterial Sirtuins
Source: Front Microbiol. 2016 May 9;7:677. doi: 10.3389/fmicb.2016.00677 (PMC4860497; doi:10.3389/fmicb.2016.00677)
Supplement: Supplementary file 1 [file Data_Sheet_1.DOC]

***Supplementary Figures***

**Phosphorylation modulates catalytic activity of mycobacterial sirtuins**

***Ghanshyam S. Yadav, Sandeep K. Ravala, Neha Malhotra, Pradip K. Chakraborti^*^***

*CSIR-Institute of Microbial Technology, Sector 39A, Chandigarh 160 036, India*

**RUNNING TITLE**

Phosphorylation of mycobacterial deacetylase

***Correspondence:*** Pradip K. Chakraborti, CSIR-Institute of Microbial Technology, Sector 39A, Chandigarh 160 036, India. *e-mail:* [pradip@imtech.res.in](mailto:pradip@imtech.res.in)

**31-44 FDPYELSS*TQGWLR Ser-38**

**
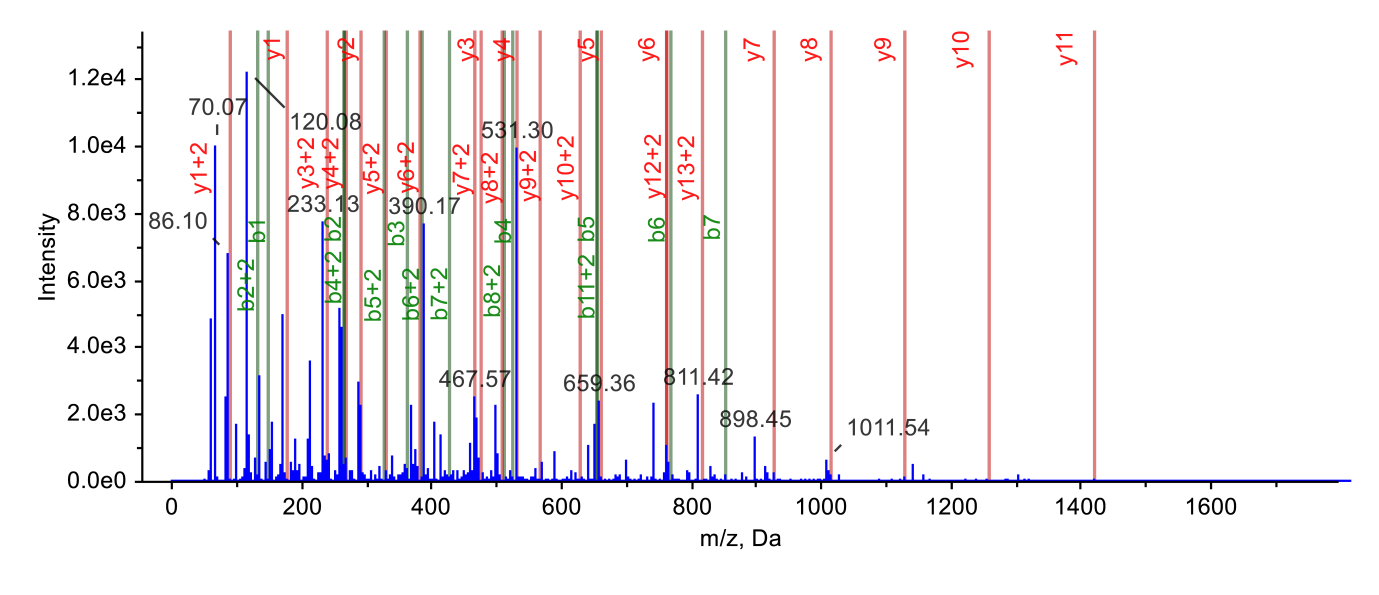
**

| Residue | b | b+2 | y | y+2 |
| --- | --- | --- | --- | --- |
| F | 148.0757 | 74.5415 | 1778.779 | 889.8929 |
| D | 263.1026 | 132.055 | 1631.71 | 816.3587 |
| P | 360.1554 | 180.5813 | 1516.683 | 758.8452 |
| Y | 523.2187 | 262.113 | 1419.63 | 710.3188 |
| E | 652.2613 | 326.6343 | 1256.567 | 628.7872 |
| L | 765.3454 | 383.1763 | 1127.525 | 564.2659 |
| S | 852.3774 | 426.6923 | 1014.44 | 507.7239 |
| S[Pho] | 1019.376 | 510.1915 | 927.4084 | 464.2078 |
| T | 1120.423 | 560.7154 | 760.41 | 380.7087 |
| Q | 1248.482 | 624.7447 | 659.3624 | 330.1848 |
| G | 1305.504 | 653.2554 | 531.3038 | 266.1555 |
| W | 1491.583 | 746.295 | 474.2823 | 237.6448 |
| L | 1604.667 | 802.8371 | 288.203 | 144.6051 |
| R | 1760.768 | 880.8876 | 175.119 | 88.0631 |
|  |  |  |  |  |

**31-44 FDPYELSS*TQGWLR Thr-39**

**
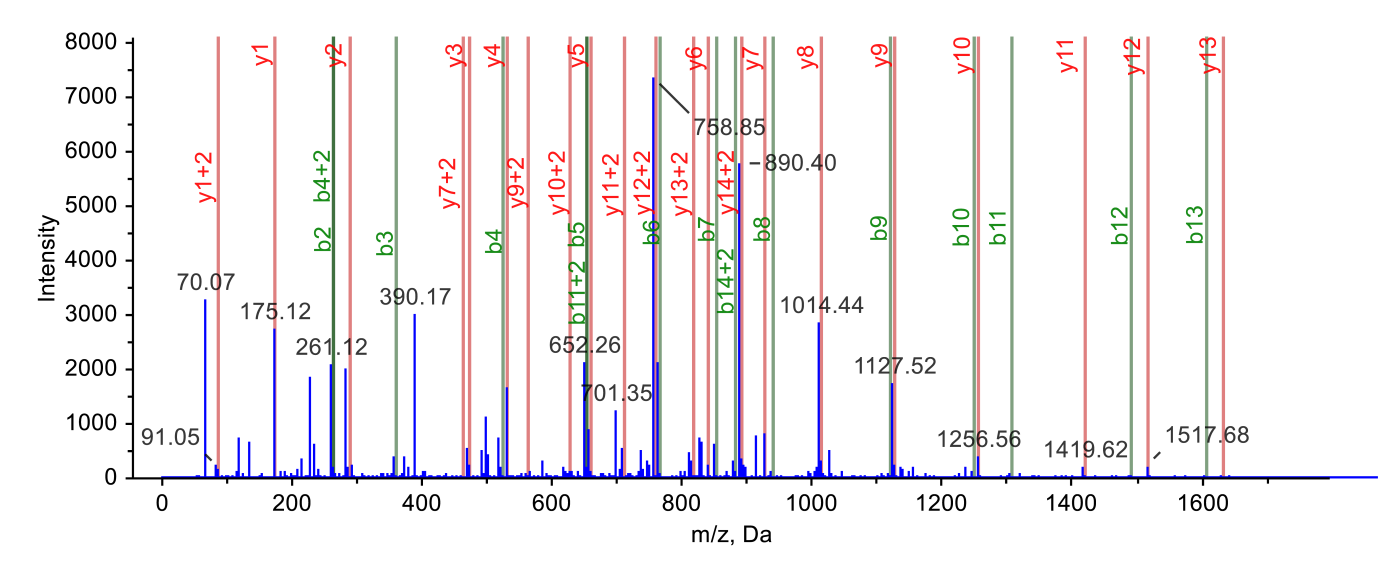
**

| Residue | b | b+2 | y | y+2 |
| --- | --- | --- | --- | --- |
| F | 148.0757 | 74.5415 | 1778.779 | 889.8929 |
| D | 263.1026 | 132.055 | 1631.71 | 816.3587 |
| P | 360.1554 | 180.5813 | 1516.683 | 758.8452 |
| Y | 523.2187 | 262.113 | 1419.63 | 710.3188 |
| E | 652.2613 | 326.6343 | 1256.567 | 628.7872 |
| L | 765.3454 | 383.1763 | 1127.525 | 564.2659 |
| S | 852.3774 | 426.6923 | 1014.44 | 507.7239 |
| S | 939.4094 | 470.2084 | 927.4084 | 464.2078 |
| T[Pho] | 1120.423 | 560.7154 | 840.3764 | 420.6918 |
| Q | 1248.482 | 624.7447 | 659.3624 | 330.1848 |
| G | 1305.504 | 653.2554 | 531.3038 | 266.1555 |
| W | 1491.583 | 746.295 | 474.2823 | 237.6448 |
| L | 1604.667 | 802.8371 | 288.203 | 144.6051 |
| R | 1760.768 | 880.8876 | 175.119 | 88.0631 |

**162-195 SAVEATGSADVMVVVGTS*AIVYPAAGLPDLALAR Ser-179**

**
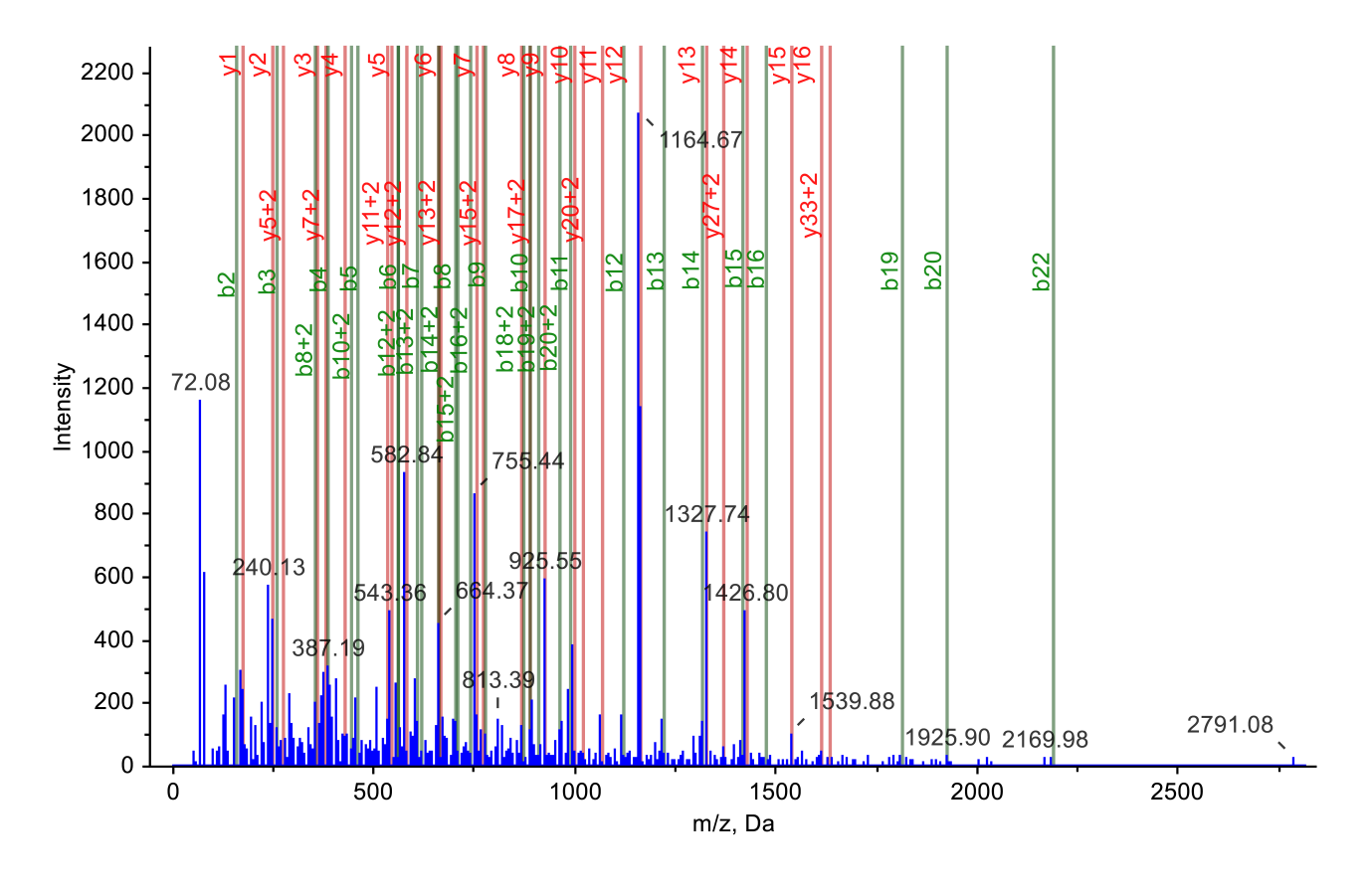
**

| Residue | b | b+2 | y | y+2 |
| --- | --- | --- | --- | --- |
| S | 88.0393 | 44.5233 | 3351.691 | 1676.349 |
| A | 159.0764 | 80.0418 | 3264.659 | 1632.833 |
| V | 258.1448 | 129.5761 | 3193.621 | 1597.314 |
| E | 387.1874 | 194.0974 | 3094.553 | 1547.78 |
| A | 458.2245 | 229.6159 | 2965.51 | 1483.259 |
| T | 559.2722 | 280.1397 | 2894.473 | 1447.74 |
| G | 616.2937 | 308.6505 | 2793.426 | 1397.217 |
| S | 703.3257 | 352.1665 | 2736.404 | 1368.706 |
| A | 774.3628 | 387.6851 | 2649.372 | 1325.19 |
| D | 889.3898 | 445.1985 | 2578.335 | 1289.671 |
| V | 988.4582 | 494.7327 | 2463.308 | 1232.158 |
| M | 1119.499 | 560.253 | 2364.24 | 1182.624 |
| V | 1218.567 | 609.7872 | 2233.199 | 1117.103 |
| V | 1317.636 | 659.3214 | 2134.131 | 1067.569 |
| V | 1416.704 | 708.8556 | 2035.062 | 1018.035 |
| G | 1473.725 | 737.3663 | 1935.994 | 968.5006 |
| T | 1574.773 | 787.8902 | 1878.973 | 939.9899 |
| S[Pho] | 1741.771 | 871.3893 | 1777.925 | 889.466 |
| A | 1812.809 | 906.9079 | 1610.927 | 805.9669 |
| I | 1925.893 | 963.4499 | 1539.889 | 770.4483 |
| V | 2024.961 | 1012.984 | 1426.805 | 713.9063 |
| Y | 2188.024 | 1094.516 | 1327.737 | 664.3721 |
| P | 2285.077 | 1143.042 | 1164.674 | 582.8404 |
| A | 2356.114 | 1178.561 | 1067.621 | 534.314 |
| A | 2427.151 | 1214.079 | 996.5837 | 498.7955 |
| G | 2484.173 | 1242.59 | 925.5465 | 463.2769 |
| L | 2597.257 | 1299.132 | 868.5251 | 434.7662 |
| P | 2694.31 | 1347.658 | 755.441 | 378.2241 |
| D | 2809.337 | 1405.172 | 658.3883 | 329.6978 |
| L | 2922.421 | 1461.714 | 543.3613 | 272.1843 |
| A | 2993.458 | 1497.233 | 430.2772 | 215.6423 |
| L | 3106.542 | 1553.775 | 359.2401 | 180.1237 |
| A | 3177.579 | 1589.293 | 246.1561 | 123.5817 |
| R | 3333.68 | 1667.344 | 175.119 | 88.0631 |

**196-218 GT*AVIEVNPEPTPLSGSATISIR Thr-197**

**
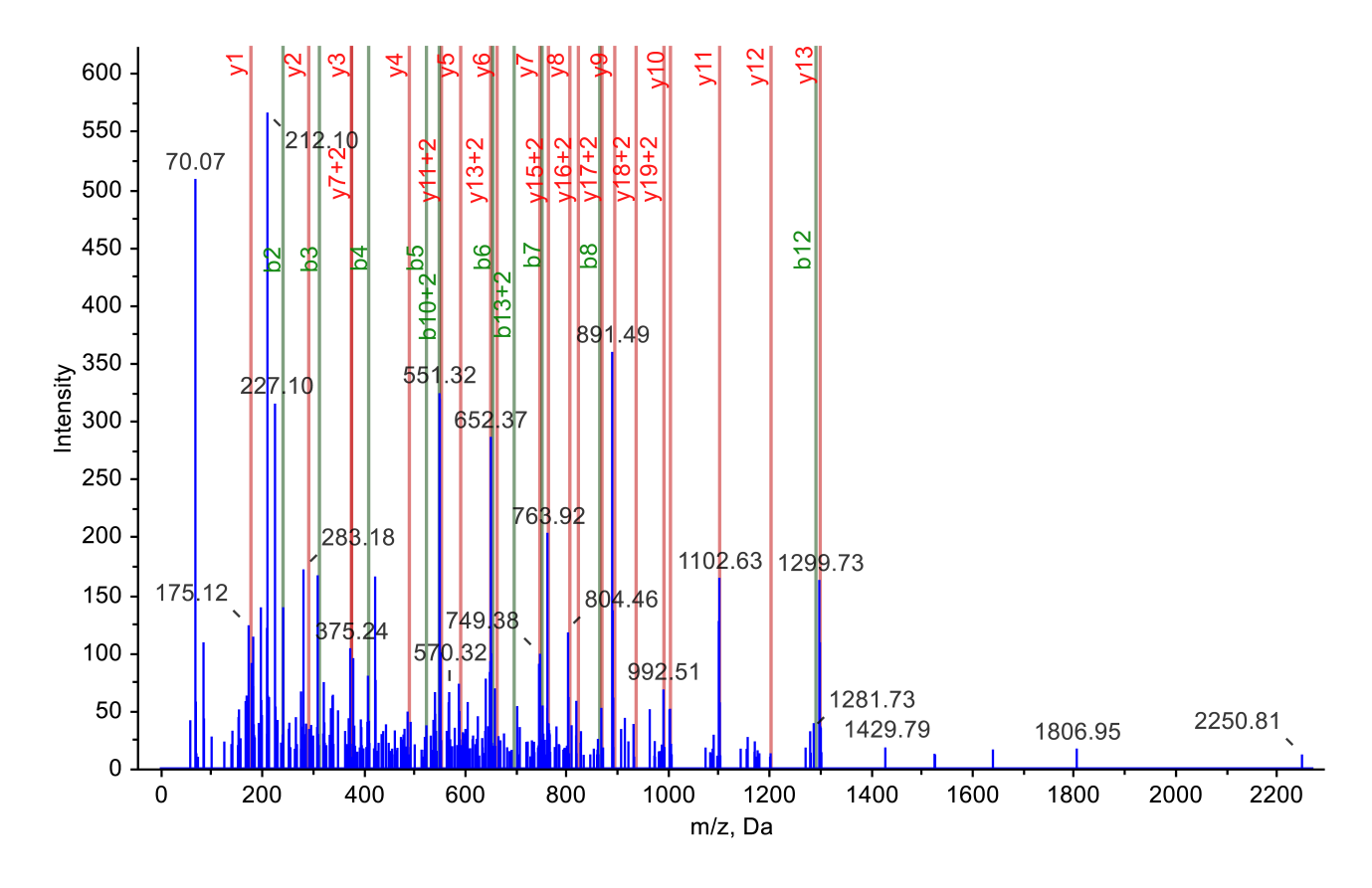
**

| Residue | b | b+2 | y | y+2 |
| --- | --- | --- | --- | --- |
| G | 58.0287 | 29.518 | 2389.201 | 1195.104 |
| T[Pho] | 239.0427 | 120.025 | 2332.18 | 1166.593 |
| A | 310.0799 | 155.5436 | 2151.166 | 1076.086 |
| V | 409.1483 | 205.0778 | 2080.129 | 1040.568 |
| I | 522.2323 | 261.6198 | 1981.06 | 991.0337 |
| E | 651.2749 | 326.1411 | 1867.976 | 934.4916 |
| V | 750.3433 | 375.6753 | 1738.933 | 869.9703 |
| N | 864.3863 | 432.6968 | 1639.865 | 820.4361 |
| P | 961.439 | 481.2232 | 1525.822 | 763.4147 |
| E | 1090.482 | 545.7445 | 1428.769 | 714.8883 |
| P | 1187.534 | 594.2708 | 1299.727 | 650.367 |
| T | 1288.582 | 644.7947 | 1202.674 | 601.8406 |
| P | 1385.635 | 693.3211 | 1101.626 | 551.3168 |
| L | 1498.719 | 749.8631 | 1004.574 | 502.7904 |
| S | 1585.751 | 793.3791 | 891.4894 | 446.2483 |
| G | 1642.772 | 821.8898 | 804.4574 | 402.7323 |
| S | 1729.804 | 865.4058 | 747.4359 | 374.2216 |
| A | 1800.842 | 900.9244 | 660.4039 | 330.7056 |
| T | 1901.889 | 951.4482 | 589.3668 | 295.187 |
| I | 2014.973 | 1007.99 | 488.3191 | 244.6632 |
| S | 2102.005 | 1051.506 | 375.235 | 188.1212 |
| I | 2215.089 | 1108.048 | 288.203 | 144.6051 |
| R | 2371.191 | 1186.099 | 175.119 | 88.0631 |

**196-218 GTAVIEVNPEPTPLSGS*ATISIR Ser-212**

**
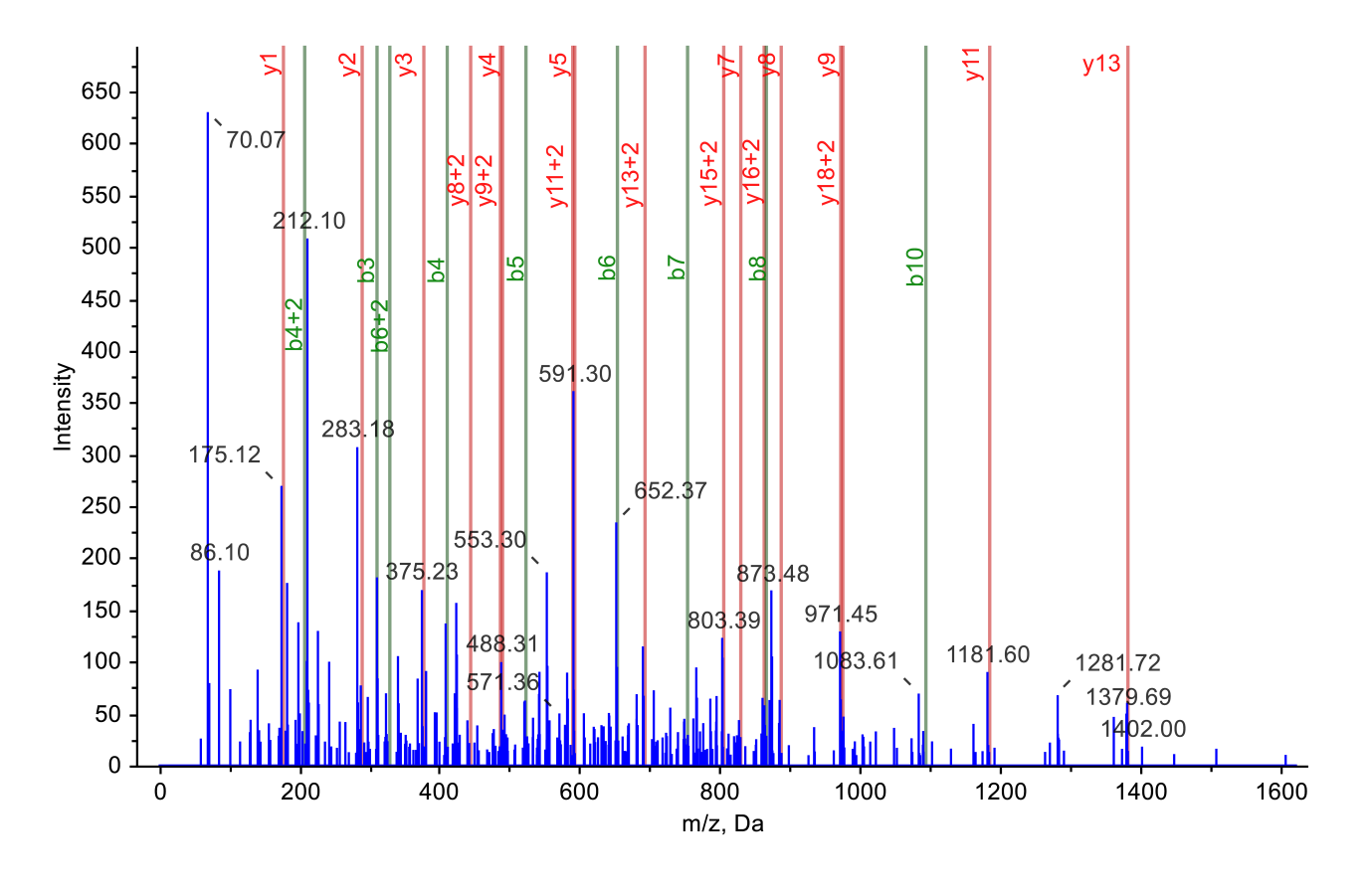
**

| Residue | b | b+2 | y | y+2 |
| --- | --- | --- | --- | --- |
| G | 58.0287 | 29.518 | 2469.167 | 1235.087 |
| T[Pho] | 239.0427 | 120.025 | 2412.146 | 1206.577 |
| A | 310.0799 | 155.5436 | 2231.132 | 1116.07 |
| V | 409.1483 | 205.0778 | 2160.095 | 1080.551 |
| I | 522.2323 | 261.6198 | 2061.026 | 1031.017 |
| E | 651.2749 | 326.1411 | 1947.942 | 974.4748 |
| V | 750.3433 | 375.6753 | 1818.9 | 909.9535 |
| N | 864.3863 | 432.6968 | 1719.831 | 860.4193 |
| P | 961.439 | 481.2232 | 1605.788 | 803.3978 |
| E | 1090.482 | 545.7445 | 1508.736 | 754.8714 |
| P | 1187.534 | 594.2708 | 1379.693 | 690.3501 |
| T | 1288.582 | 644.7947 | 1282.64 | 641.8238 |
| P | 1385.635 | 693.3211 | 1181.593 | 591.2999 |
| L | 1498.719 | 749.8631 | 1084.54 | 542.7735 |
| S | 1585.751 | 793.3791 | 971.4558 | 486.2315 |
| G | 1642.772 | 821.8898 | 884.4237 | 442.7155 |
| S[Pho] | 1809.771 | 905.389 | 827.4023 | 414.2048 |
| A | 1880.808 | 940.9076 | 660.4039 | 330.7056 |
| T | 1981.856 | 991.4314 | 589.3668 | 295.187 |
| I | 2094.94 | 1047.973 | 488.3191 | 244.6632 |
| S | 2181.972 | 1091.49 | 375.235 | 188.1212 |
| I | 2295.056 | 1148.032 | 288.203 | 144.6051 |
| R | 2451.157 | 1226.082 | 175.119 | 88.0631 |

**196-218 GTAVIEVNPEPTPLSGSAT*ISIR Thr-214**

**
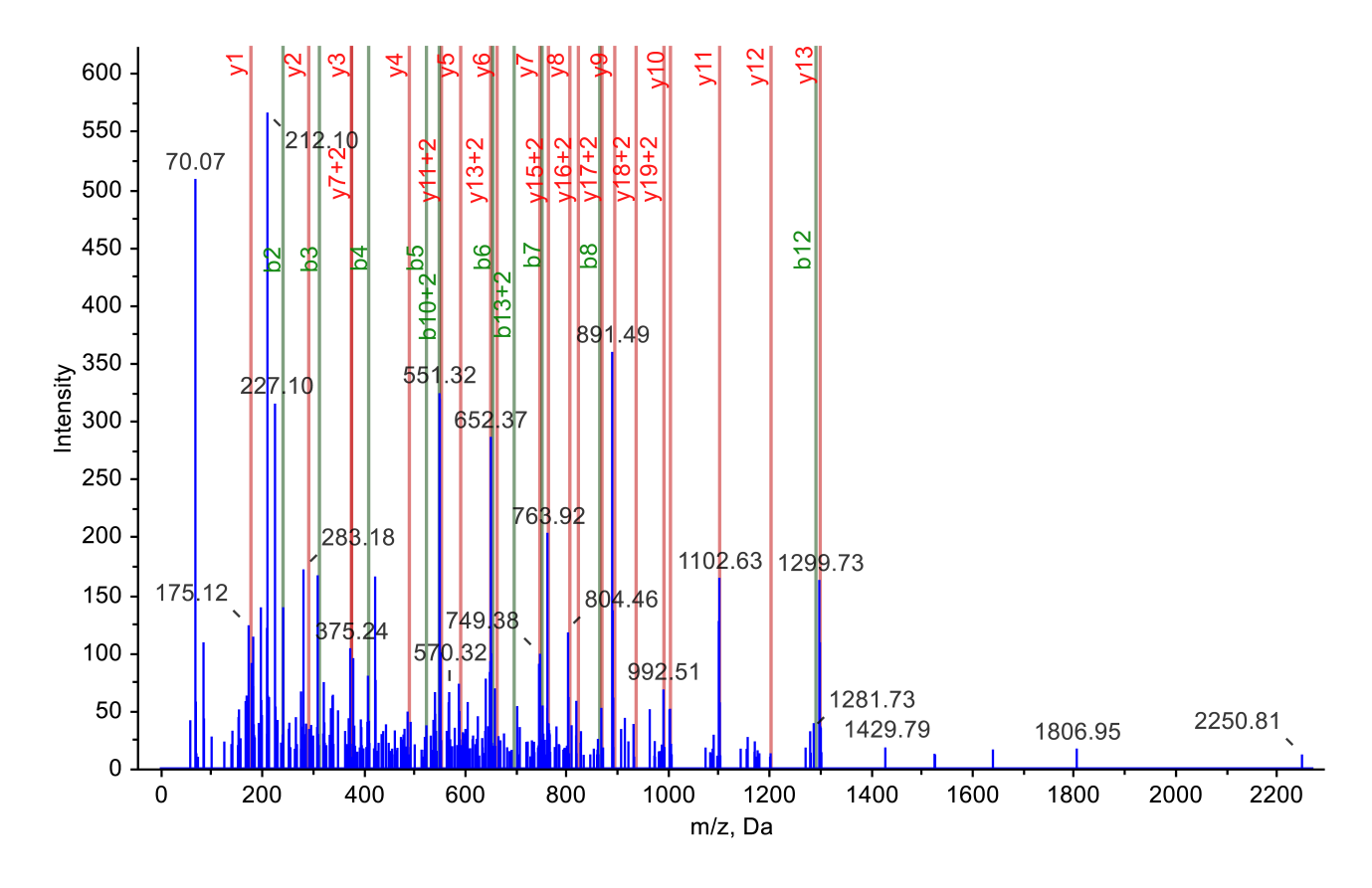
**

| Residue | b | b+2 | y | y+2 |
| --- | --- | --- | --- | --- |
| G | 58.0287 | 29.518 | 2389.201 | 1195.104 |
| T[Pho] | 239.0427 | 120.025 | 2332.18 | 1166.593 |
| A | 310.0799 | 155.5436 | 2151.166 | 1076.086 |
| V | 409.1483 | 205.0778 | 2080.129 | 1040.568 |
| I | 522.2323 | 261.6198 | 1981.06 | 991.0337 |
| E | 651.2749 | 326.1411 | 1867.976 | 934.4916 |
| V | 750.3433 | 375.6753 | 1738.933 | 869.9703 |
| N | 864.3863 | 432.6968 | 1639.865 | 820.4361 |
| P | 961.439 | 481.2232 | 1525.822 | 763.4147 |
| E | 1090.482 | 545.7445 | 1428.769 | 714.8883 |
| P | 1187.534 | 594.2708 | 1299.727 | 650.367 |
| T | 1288.582 | 644.7947 | 1202.674 | 601.8406 |
| P | 1385.635 | 693.3211 | 1101.626 | 551.3168 |
| L | 1498.719 | 749.8631 | 1004.574 | 502.7904 |
| S | 1585.751 | 793.3791 | 891.4894 | 446.2483 |
| G | 1642.772 | 821.8898 | 804.4574 | 402.7323 |
| S | 1729.804 | 865.4058 | 747.4359 | 374.2216 |
| A | 1800.842 | 900.9244 | 660.4039 | 330.7056 |
| T | 1901.889 | 951.4482 | 589.3668 | 295.187 |
| I | 2014.973 | 1007.99 | 488.3191 | 244.6632 |
| S | 2102.005 | 1051.506 | 375.235 | 188.1212 |
| I | 2215.089 | 1108.048 | 288.203 | 144.6051 |
| R | 2371.191 | 1186.099 | 175.119 | 88.0631 |

**219-231 ESAS*QALPGLLER Ser-222**

**
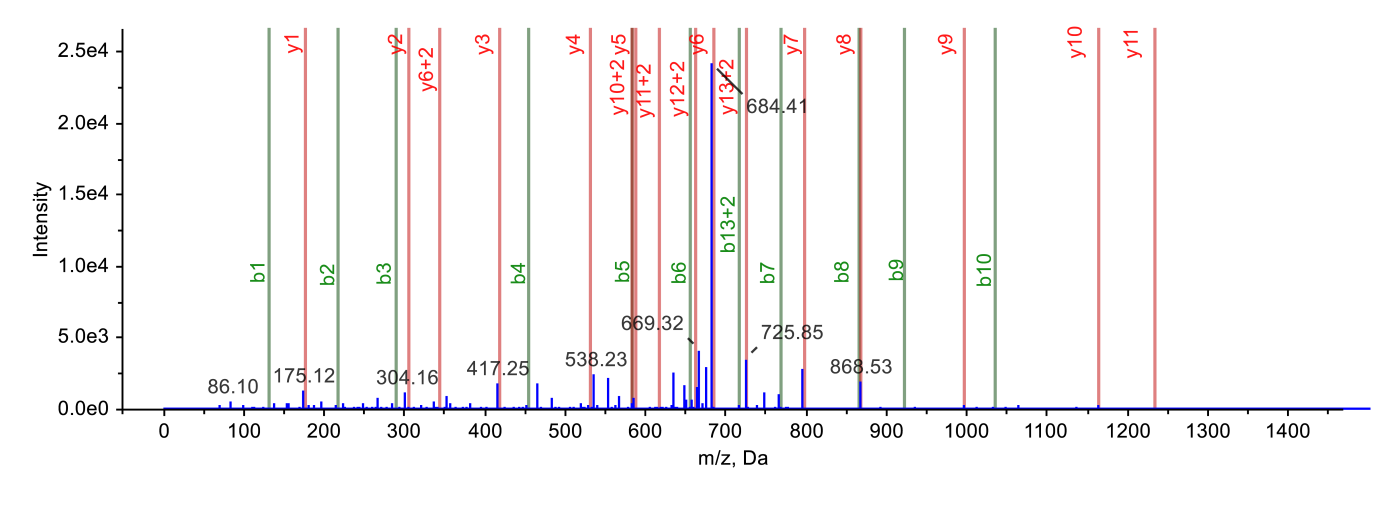
**

| Residue | b | b+2 | y | y+2 |
| --- | --- | --- | --- | --- |
| E | 130.0499 | 65.5286 | 1450.694 | 725.8505 |
| S | 217.0819 | 109.0446 | 1321.651 | 661.3292 |
| A | 288.119 | 144.5631 | 1234.619 | 617.8132 |
| S[Pho] | 455.1174 | 228.0623 | 1163.582 | 582.2946 |
| Q | 583.1759 | 292.0916 | 996.5837 | 498.7955 |
| A | 654.2131 | 327.6102 | 868.5251 | 434.7662 |
| L | 767.2971 | 384.1522 | 797.488 | 399.2476 |
| P | 864.3499 | 432.6786 | 684.4039 | 342.7056 |
| G | 921.3714 | 461.1893 | 587.3511 | 294.1792 |
| L | 1034.455 | 517.7313 | 530.3297 | 265.6685 |
| L | 1147.54 | 574.2734 | 417.2456 | 209.1264 |
| E | 1276.582 | 638.7947 | 304.1615 | 152.5844 |
| R | 1432.683 | 716.8452 | 175.119 | 88.0631 |

**FIGURE S1 | LC-MS/MS analysis of p-mDAC.** Protein pilot data of mass spectrometry analyses presented in **Table 1**. Fragmentation spectrum for each modified peptide following LC-MS/MS are presented (‘b’ and ‘y’ ions are displayed in green). Asterisks in each peptide represent modified residues.

**
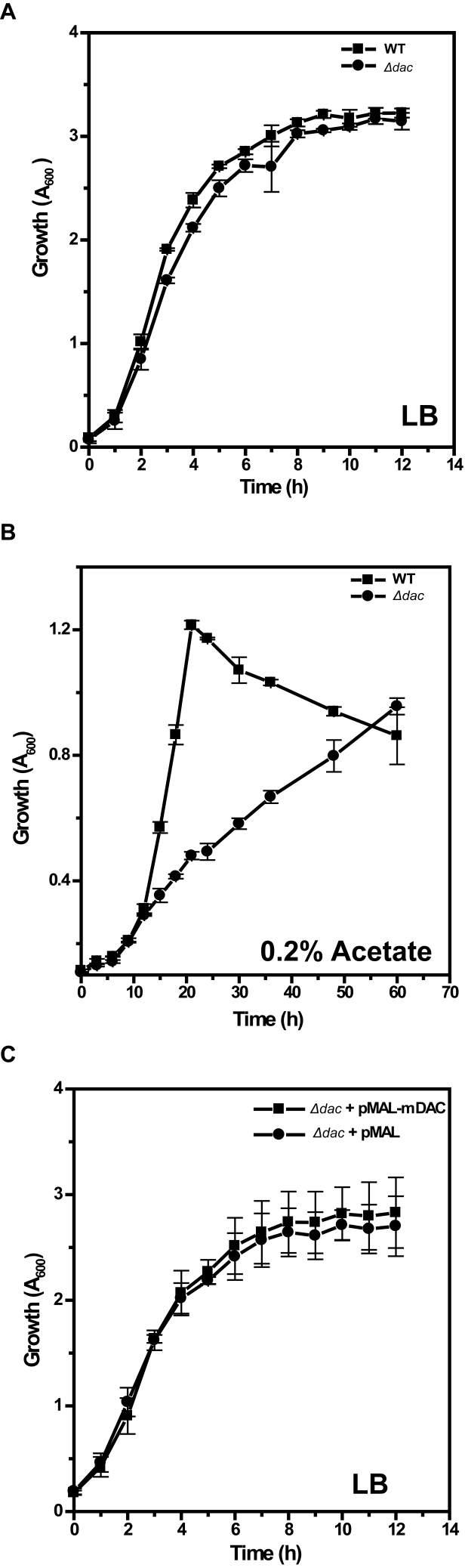
**

**FIGURE S2 | Growth curve (A_600_) of different *E. coli* cultures. (A)** Wild-type and Δdac cultures in LB media (data shown in **Figure 5A**).  **(B)** Wild-type and Δdac cultures in acetate media (data shown in **Figure 5A**). **(C)** Cultures of Δdac completed with either vector (pMAL-c2) or mDAC (pMAL-mDAC). Growth curve of the data shown in **Figure 5B** (inset; Top). Notation used: LB, Luria-Bertani broth.

***M. tuberculosis* (mDAC) and Human Sirt1**

M.tuberculosis ------------------------------------------------------------

Sirt1 MADEAALALQPGGSPSAAGADREAASSPAGEPLRKRPRRDGPGLERSPGEPGGAAPEREV

M.tuberculosis ------------------------------------------------------------

Sirt1 PAAARGCPGAAAAALWREAEAEAAAAGGEQEAQATAAAGEGDNGPGLQGPSREPPLADNL

M.tuberculosis ------------------------------------------------------------

Sirt1 YDEDDDDEGEEEEEAAAAAIGYRDNLLFGDEIITNGFHSCESDEEDRASHASSSDWTPRP

M.tuberculosis ------------------------------------------------------------

Sirt1 RIGPYTFVQQHLMIGTDPRTILKDLLPETIPPPELDDMTLWQIVINILSEPPKRKKRKDI

M.tuberculosis -------------MRVAVLSGAGISAESGVPTFRDDKNGLWARFDPY--------ELSST

Sirt1 NTIEDAVKLLQECKKIIVLTGAGVSVSCGIPDFRSR-DGIYARLAVDFPDLPDPQAMFDI

:: **:***:*...*:* **. :*::**: : .

M.tuberculosis QGWLRNPERVWGWYLWRHYLVANVEPNDGHRAIAAWQ-DHAEVSVITQNVDDLHERAGSG

Sirt1 EYFRKDPRPFFKFA--KEIYPGQFQPSLCHKFIALSDKEGKLLRNYTQNIDTLEQVAGIQ

: : ::*. .: : :. .:.:*. *: ** : : : ***:* *.: **

M.tuberculosis AVHHLHGSLFEFRCARCGVPYT----------DALPEMPEPAIEVEPPVCDCGGLIRPDI

Sirt1 RIIQCHGSFATASCLICKYKVDCEAVRGDIFNQVVPRCPR------CPADEPLAIMKPEI

: : ***: * * :.:*. *. *. : .:::*:*

M.tuberculosis VWFGEPLPEEPWRSAVEATGSADVMVVVGTSAIVYPAAGLPDLALARGTAVIEVNPEPTP

Sirt1 VFFGENLPEQFHRAMKYDKDEVDLLIVIGSSLKVRPVALIPSSIP-HEVPQILINREPLP

*:*** ***: *: . ..*:::*:*:* * *.* :*. : . * :* ** *

M.tuberculosis LS-------GSA**T**ISIRESASQALPGLL--------------ERLPALLK----------

Sirt1 HLHFDVELLGDCDVIINELC-HRLGGEYAKLCCNPVKLSEITEKPPRTQKELAYLSELPP

*.. : *.* . : * * *: * *

M.tuberculosis ------------------------------------------------------------

Sirt1 TPLHVSEDSSSPERTSPPDSSVIVTLLDQAAKSNDDLDVSESKGCMEEKPQEVQTSRNVE

M.tuberculosis ------------------------------------------------------------

Sirt1 SIAEQMENPDLKNVGSSTGEKNERTSVAGTVRKCWPNRVAKEQISRRLDGNQYLFLPPNR

M.tuberculosis ------------------------------------------------------------

Sirt1 YIFHGAEVYSDSEDDVLSSSSCGSNSDSGTCQSPSLEEPMEDESEIEEFYNGLEDEPDVP

M.tuberculosis --------------------------------------

Sirt1 ERAGGAGFGTDGDDQEAINEAISVKQEVTDMNYPSNKS

***M. tuberculosis* (mDAC) and Human Sirt2**

M.tuberculosis ------------------------------------------------------------

Sirt2 MAEPDPSHPLETQAGKVQEAQDSDSDSEGGAAGGEADMDFLRNLFSQTLSLGSQKERLLD

M.tuberculosis ----------------MRVAVLSGAGISAESGVPTFRDDKNGLWARFDPYELSSTQGWL-

Sirt2 ELTLEGVARYMQSERCRRVICLVGAGISTSAGIPDFRSPSTGLYDNLEKYHLPYPEAIFE

** * *****:.:*:* **. ..**: .:: *.* :. :

M.tuberculosis -----RNPERVWGWYLWRHYLVANVEPNDGHRAIAAWQDH-AEVSVITQNVDDLHERAGS

Sirt2 ISYFKKHPEPFFAL--AKELYPGQFKPTICHYFMRLLKDKGLLLRCYTQNIDTLERIAGL

:.** .:. :. .:.:*. * : :*: : ***:* *.. **

M.tuberculosis G--AVHHLHGSLFEFRCARCGVPYTDALPEMPEPA-IEVEPPVCDCGGLIRPDIVWFGEP

Sirt2 EQEDLVEAHGTFYTSHCVSASCRHEYPLSWMKEKIFSEVTPKCEDCQSLVKPDIVFFGES

: . **::: :*. .. : * * * ** * ** .*::****:***

M.tuberculosis LPEEPWRSAVEATGSADVMVVVGTSAIVYPAAGLPDLALARGTAVIEVNPE----PTPLS

Sirt2 LPARFFSCMQSDFLKVDLLLVMGTSLQVQPFASLISKAPLSTPRL-LINKEKAGQSDPFL

** . : . . ..*:::*:*** * * *.* . * : :* * *:

M.tuberculosis G-------SA**T**ISIRE------------SASQA---LPGLLERLPALLK-----------

Sirt2 GMIMGLGGGMDFDSKKAYRDVAWLGECDQGCLALAELLGWKKELEDLVRREHASIDAQSG

* . :. :: ... * * * :.* *::

M.tuberculosis --------------------------------

Sirt2 AGVPNPSTSASPKKSPPPAKDEARTTEREKPQ

***M. tuberculosis* (mDAC) and Human Sirt3**

M.tuberculosis ------------------------------------------------------------

Sirt3 MAFWGWRAAAALRLWGRVVERVEAGGGVGPFQACGCRLVLGGRDDVSAGLRGSHGARGEP

M.tuberculosis ------------------------------------------------------------

Sirt3 LDPARPLQRPPRPEVPRAFRRQPRAAAPSFFFSSIKGGRRSISFSVGASSVVGSGGSSDK

M.tuberculosis -----------------MRVAVLSGAGISAESGVPTFRDDKNGLWARFDPYELSSTQGWL

Sirt3 GKLSLQDVAELIRARACQRVVVMVGAGISTPSGIPDFRSPGSGLYSNLQQYDLPYPEAIF

**.*: *****: **:* **. .**::.:: *:* :. :

M.tuberculosis RNP------ERVWGWYLWRHYLVANVEPNDGHRAIAAWQDH-AEVSVITQNVDDLHERAG

Sirt3 ELPFFFHNPKPFFT--LAKELYPGNYKPNVTHYFLRLLHDKGLLLRLYTQNIDGLERVSG

. * : .: * :. .* :** * : :*: : : ***:* *.. :*

M.tuberculosis SG--AVHHLHGSLFEFRCARCGVPYTDALPEMPEPAIEVEPPVCDCGGLIRPDIVWFGEP

Sirt3 IPASKLVEAHGTFASATCTVCQRPFPGEDIRA-DVMADRVPRCPVCTGVVKPDIVFFGEP

: . **:: . *: * *: . : : * * *:::****:****

M.tuberculosis LPEEPWRSAVEATGSADVMVVVGTSAIVYPAAGLPDLALARGTAVIEVNPEPTPLSGSA**T**

Sirt3 LPQRFLLHVVD-FPMADLLLILGTSLEVEPFASLTEAVRSSVPRLL-INR---DLVGPLA

**:. .*: **:::::*** * * *.* : . : :: :* * * :

M.tuberculosis ISIRESASQA-------------LPGLLERLPALLK-----------

Sirt3 WHPRSRDVAQLGDVVHGVESLVELLGWTEEMRDLVQRETGKLDGPDK

*. * * *.: *::

***M. tuberculosis* (mDAC) and Human Sirt4**

M.tuberculosis ------------------------------------------------------MRVAVL

Sirt4 MKMSFALTFRSAKGRWIANPSQPCSKASIGLFVPASPPLDPEKVKELQRFITLSKRLLVM

*: *:

M.tuberculosis SGAGISAESGVPTFRDDKNGLWARFDPYELSSTQGWLRNP--ERVWGWYLWRHYLVANVE

Sirt4 TGAGISTESGIPDYRSEKVGLYARTDRRPIQHGDFVRSAPIRQRYWARNFVGWPQFSSHQ

:*****:***:* :*.:* **:** * :. : * :* *. : .:. :

M.tuberculosis PNDGHRAIAAWQDHAEV-SVITQNVDDLHERAGSGAVHHLHGSLFEFRCARCGVPYTDAL

Sirt4 PNPAHWALSTWEKLGKLYWLVTQNVDALHTKAGSRRLTELHGCMDRVLCLDCGEQTPRGV

** .* *:::*:. .:: ::***** ** :*** : .***.: .. * ** .:

M.tuberculosis PEMP--------------------------EPAIEVEPPVCDCGGLIRPDIVWFGEPLPE

Sirt4 LQERFQVLNPTWSAEAHGLAPDGDVFLSEEQVRSFQVPTCVQCGGHLKPDVVFFGDTVNP

: : * :*** ::**:*:**: :

M.tuberculosis EPWRSAVEATGSADVMVVVGTSAIVYPAAGLPDLALARGTAVIEVNPEPTPLSGSA**T**ISI

Sirt4 DKVDFVHKRVKEADSLLVVGSSLQVYSGYRFILTAWEKKLPIAILNIGPTRSDDLACLKL

: . : . .** ::***:* ** . : * : : :* ** . * :.:

M.tuberculosis RESASQALPGLLERLPALLK

Sirt4 NSRCGELLPLI-DPC-----

.. ..: ** : :

***M. tuberculosis* (mDAC) and Human Sirt5**

M.tuberculosis --------------------------------------------------MRVAVLSGAG

Sirt5 MRPLQIVPSRLISQLYCGLKPPASTRNQICLKMARPSSSMADFRKFFAKAKHIVIISGAG

::.::****

M.tuberculosis ISAESGVPTFRDDKNGLWARFDPYELSSTQGWLRNPERVWGWYLWRHYLVANVEPNDGHR

Sirt5 VSAESGVPTFRG-AGGYWRKWQAQDLATPLAFAHNPSRVWEFYHYRREVMGSKEPNAGHR

:********** * * ::: :*:: .: :**.*** :* :*: ::.. *** ***

M.tuberculosis AIAAWQD-----HAEVSVITQNVDDLHERAGSGAVHHLHGSLFEFRCARCGVPYTDALPE

Sirt5 AIAECETRLGKQGRRVVVITQNIDELHRKAGTKNLLEIHGSLFKTRCTSCGVVAENYKSP

*** : .* *****:*:**.:**: : .:*****: **: *** :

M.tuberculosis ---------MPEPA-----IEV----EPPVCDCGGLIRPDIVWFGEPLPEEPWRSAVEAT

Sirt5 ICPALSGKGAPEPGTQDASIPVEKLPRCEEAGCGGLLRPHVVWFGENLDPAILEEVDREL

***. * * . . ****:**.:***** * ... .

M.tuberculosis GSADVMVVVGTSAIVYPAAGLPDLALARGTAVIEVNPEPTPLSGSA**T**ISIRESASQALPG

Sirt5 AHCDLCLVVGTSSVVYPAAMFAPQVAARGVPVAEFNTETTPATNRFRFHFQGPCGTTLPE

. .*: :*****::***** : . ***. * *.* * ** : : :: .. :**

M.tuberculosis LLERLPALLK-

Sirt5 ALACHENETVS

*

***M. tuberculosis* (mDAC) and Human Sirt6**

M.tuberculosis --------------------------------------------MRVAVLSGAGISAESG

Sirt6 MSVNYAAGLSPYADKGKCGLPEIFDPPEELERKVWELARLVWQSSSVVFHTGAGISTASG

*.. :*****: **

M.tuberculosis VPTFRDDKNGLWARFDPYELSSTQGWLRNPERVWGWYLWRHYLVANVEPNDGHRAIAAWQ

Sirt6 IPDFRGP-HGVWTMEERGLAPKF-----------------DTTFESARPTQTHMALVQLE

:* ** .*:*: : . . . ...*.: * *:. :

M.tuberculosis DH-AEVSVITQNVDDLHERAGS--GAVHHLHGSLFEFRCARCGVPYTDALPEMP--E---

Sirt6 RVGLLRFLVSQNVDGLHVRSGFPRDKLAELHGNMFVEECAKCKTQYVRDTVVGTMGLKAT

:::**** ** *:* : .***.:* .**:* . *.

M.tuberculosis ---PAIEVEPPVCDCGGLIRPDIVWFGEPLPEEPWRSAVEATGSADVMVVVGTSAIVYPA

Sirt6 GRLCTVAKARGLRACRGELRDTILDWEDSLPDRDLALADEASRNADLSITLGTSLQIRPS

:: : * * :* *: : : **:. * **: .**: :.:*** : *:

M.tuberculosis AGLPDLALARGTAVIEVNPEPTPLSGSA**T**ISIRESASQALPGLLERLPALLK--------

Sirt6 GNLPLATKRRGGRLVIVNLQPTKHDRHADLRIHGYVDEVMTRLMKHLGLEIPAWDGPRVL

. ** : ** :: ** :** . * : *: ..:.: *:::* :

M.tuberculosis ------------------------------------------------------------

Sirt6 ERALPPLPRPPTPKLEPKEESPTRINGSIPAGPKQEPCAQHNGSEPASPKRERPTSPAPH

M.tuberculosis -------------

Sirt6 RPPKRVKAKAVPS

***M. tuberculosis* (mDAC) and Human Sirt7**

M.tuberculosis ------------------------------------------------------------

Sirt7 MAAGGLSRSERKAAERVRRLREEQQRERLRQVSRILRKAAAERSAEEGRLLAESADLVTE

M.tuberculosis ---------------------------------------MRVAVLSGAGISAESGVPTFR

Sirt7 LQGRSRRREGLKRRQEEVCDDPEELRGKVRELASAVRNAKYLVVYTGAGISTAASIPDYR

:.* :*****: :.:* :*

M.tuberculosis DDKNGLWARFDPYELSSTQGWLRNPERVWGWYLWRHYLVANVEPNDGHRAIAAWQD-HAE

Sirt7 G-PNGVWTLLQKGRSVSAAD------------------LSEAEPTLTHMSITRLHEQKLV

**:*: :: . *: :::.**. * :*: :: :

M.tuberculosis VSVITQNVDDLHERAGS--GAVHHLHGSLFEFRCARCGVPY--TDALPEMPEP---AIEV

Sirt7 QHVVSQNCDGLHLRSGLPRTAISELHGNMYIEVCTSCVPNREYVRVFDVTERTALHRHQT

*::** * ** *:* *: .***.:: *: * . .: . :.

M.tuberculosis EPPVCDCGGLIRPDIVWFGEPLP---EEPWRSAVEATGSADVMVVVGTSAIVYPAAGLPD

Sirt7 GRTCHKCGTQLRDTIVHFGERGTLGQPLNWEAATEAASRADTILCLGSSLKVLKKY--PR

.** :* ** *** *.:*.**:. **.:: :*:* * *

M.tuberculosis LA-----LARGTAVIEVNPEPTPLSGSA**T**ISIRESASQALPGLLE----RLPALLK----

Sirt7 LWCMTKPPSRRPKLYIVNLQWTPKDDWAALKLHGKCDDVMRLLMAELGLEIPAYSRWQDP

* :* : ** : ** . *::.:: ...:.: *: .:** :

M.tuberculosis ------------------------------------------------------------

Sirt7 IFSLATPLRAGEEGSHSRKSLCRSREEAPPGDRGAPLSSAPILGGWFGRGCTKRTKRKKV

M.tuberculosis -

Sirt7 T

***M. tuberculosis* (mDAC) and Gram positive bacteria**

M.tuberculosis ----------------MRVAVLSGAGISAESGVPTFRDDKNGLWARFD-----PYELSST

B.cereus MFVQQYEEVRSILEKAKKITVLTGAGASTESGIPDFRS-SNGLYADAN-----VEMYLSR

S.aureus -MNTQIQQLKNIIDSSQNITFFTGAGISVASGIPDFRS-LGGLYDQISENGYSPEYLLST

L.monocytogenes -----MNKLNEALKKAERIVFLTGAGVSVPSGIPDYRS-KNGLYAGMS----SPEYMLSH

.:..::*** *. **:* :*. **: . *

M.tuberculosis QGWLRNPERVWGWY--LWRHYLVANVEPNDGHRAIAAWQD-HAEVSVITQNVDDLHERAG

B.cereus GYYNRNPKEFWKHYKEIFQINTFHQYKANRGHRFLAELEEQGKDITILTQNIDGLHQVGG

S.aureus DYLQSDPKGFMDFC---FQYLLFTDKQPNIVHQWIAQLEKDHRSLGVITQNIDGLHSNAG

L.monocytogenes TCLVREPEKFYQFV---TENMYYPNAVPNVIHKKMAEIETE-KDVTIITQNIDGLHEKAG

:*: . . : * *: :* : .: ::***:* **. .*

M.tuberculosis SGAVHHLHGSLFEFRCARCGVPYTDALPEMPEPAIEVE-PPVCDCGGLIRPDIVWFGEPL

B.cereus SKHVIDLHGTLQTAHCPKCKAGYDLQFM------IDHEVPRCEKCNFILNPDVVLYGDTL

S.aureus SLNTDELHGTLNRFYCTKCQQTYTKSYV------IDNHLHHCESCGHTIRPDIVLYGEML

L.monocytogenes SKKVVNFHGSLYHCYCQNCGMTVTAKDY------LKSDIH--TDCGGVIRPDVVLYEEAI

* . .:**:* * .* :. . .* :.**:* : : :

M.tuberculosis PEEPWRSAVEATGSADVMVVVGTSAIVYPAAGLPDLALARGTA-VIEVNPEPTPLSGSA**T**

B.cereus PQY--QNAIKRLYETDVLLVMGTSLKVQPVASFPEIAKREVGATTILVNEELTGQEYNF**D**

S.aureus DQETIFNALNKIREADTLVVLGSSLLVQPAAGLISNFE--GQNL-VIINKDETPYDTTA**N**

L.monocytogenes SESAIDQSLTAIRKADLIVIVGTSFRVSPFCNLTDYRN--KKARIFAVNKERISLPYPF**E**

: .:: .:* ::::*:* * * . : . . :* :

M.tuberculosis ISIRESASQALPGLLERLPALLK

B.cereus YVFQQKIGEFVEGLSSKE-----

S.aureus IVIHDDMVAVVQELLG-------

L.monocytogenes -MMESDAVKVFAEI---------

:... . :

**FIGURE S3 | Sequence comparison of mDAC. mDAC sequence was aligned (1:1 pair-wise alignment) with human sirtuins (Sirt1-Sirt7) and NAD^+^ dependent deacetylases from few gram-positive bacteria using Clustal Omega program. NAD^+^ binding sites are highlighted in yellow and conserved threonine residue (Thr-214) is marked in red. Notations used: M. tuberculosis, *Mycobacterium tuberculosis*; B.cereus, *Bacillus cereus*; S.aureus, *Staphylococcus aureus*; L.monocytogenes, *Listeria monocytogenes*.**


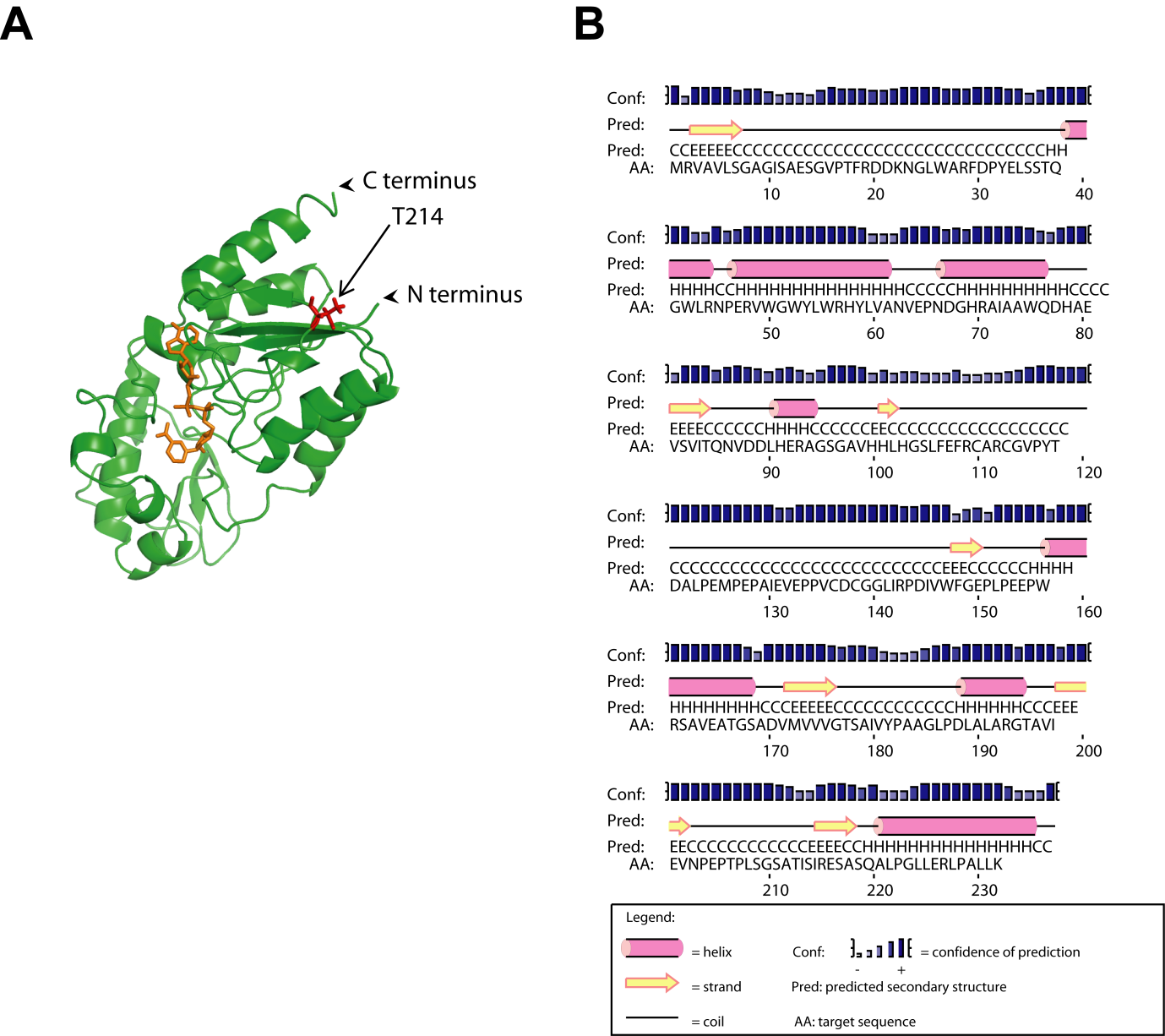


**FIGURE S4 | Modelled structure of mDAC. (A) Structure predicted using I-TASSER server. Model is based on available crystal structure of deacetylase from** *Archaeoglobus fulgidus* (PDB ID: 1ICI)*.* Position of Thr-214 and NAD^+^ (after aligning with 1ICI) are indicated in red and orange respectively. **(B)** Secondary structure prediction of mDAC using PSIPRED.
